# Dynamic Event-Triggered Control for Unmanned Aerial Vehicle Swarm Adaptive Target Enclosing Mission

**DOI:** 10.3390/s26020655

**Published:** 2026-01-18

**Authors:** Wanjing Zhang, Xinli Xu

**Affiliations:** School of Information and Control Engineering, Qingdao University of Technology, Qingdao 266520, China; xuxinli@qut.edu.cn

**Keywords:** UAV formation control, target enclosing control, event-triggered control, formation transformation

## Abstract

Multi-UAV (unmanned aerial vehicle) target enclosing control is one of the key technologies for achieving cooperative tasks. It faces limitations in communication resources and task framework separation. To address this, a distributed cooperative control strategy is proposed based on dynamic time-varying formation description and event-triggering mechanism. Firstly, a formation description method based on a geometric transformation parameter set is established to uniformly describe the translation, rotation, and scaling movements of the formation, providing a foundation for time-varying formation control. Secondly, a cooperative architecture for adaptive target enclosing tasks is designed. This architecture achieves an organic combination of formation control and target enclosing in a unified framework, thereby meeting flexible transitions between multiple formation patterns such as equidistant surrounding and variable-distance enclosing. Thirdly, a distributed dynamic event-triggered cooperative enclosing controller is developed. This strategy achieves online adjustment of communication thresholds through internal dynamic variables, significantly reducing communication while strictly ensuring system performance. By constructing a Lyapunov function, the stability and Zeno free behavior of the closed-loop system are proven. The simulation results verify this strategy, showing that this strategy can significantly reduce communication frequency while ensuring enclosing accuracy and formation consistency and effectively adapt to uniform and maneuvering target scenarios.

## 1. Introduction

UAV swarm system, with its advantages of distribution, redundancy, and collaboration, has shown broad application prospects in regional reconnaissance [1,2], disaster rescue [3,4], and environmental monitoring [5]. As one of the core directions for cooperative control of UAV swarms, the formation target enclosing task requires UAV swarms to achieve cooperative tracking and enclosing of moving targets in a dynamic environment, which puts high demands on the autonomy, collaboration, and robustness of the control system [6,7].

Many theoretical frameworks have been presented for formation control. The behavior-based approach designs a series of simple local behavior rules for each agent, relying on local interactions between individuals to generate global formation behavior [8,9].

These methods have the advantages of distribution and strong robustness. By conceptualizing the formation as a virtual rigid structure, this method maintains the desired geometry by directing each agent to follow its assigned reference point within that structure. It is suitable for tasks requiring collective maneuvers and strict formation maintenance [10,11]. Another widely used method is the leader–follower method. It establishes a clear hierarchical relationship between intelligent agents: the leader is responsible for planning or tracking the overall trajectory, and the followers maintain their formation based on preset relative states (such as distance and azimuth) with the leader or other neighbors [12,13,14]. This method has a clear structure and is easy for distributed control. Although the effectiveness of the above methods has been validated in specific scenarios, their inherent design approach still faces many challenges when dealing with integrated problems that require coupling formation control with other complex tasks such as dynamic target enclosing.

Target enclosing control aims to enable multiple UAVs to form and maintain a stable internal configuration in a dynamic environment while also coordinating as a whole to follow the trajectory of the target. This ability is a key foundation for efficient execution of subsequent tasks such as continuous monitoring or cooperative strikes in the cluster. A geometric enclosing strategy based on a preset spherical structure was proposed to achieve distributed enclosing of fast targets by multi-UAVs [15]. By defining a cooperative control term based on self-organizing behavior, a distributed constant-speed target enclosing control strategy was designed to eliminate the dependence on formation parameters [16]. A cooperative enclosing guidance protocol was designed that enables relative distance maintenance and phase adjustment, enabling the quadcopter UAVs to move cooperatively along a circular trajectory with desired angular spacing [17]. By combining backstepping control with adaptive dynamic programming technology, an optimal surround control strategy was proposed to achieve the fixed distance enclosing of targets by multi-wheeled mobile robots [18]. However, these methods often restrict agents to a fixed enclosing radius, limiting adaptability in dynamic or cluttered environments. Moreover, they typically treat formation control and target enclosing as separate problems, leading to increased systemic complexity and potential instability during task transitions. Thus, developing an integrated framework that unifies formation maneuvering with adaptive enclosing is imperative for versatile and reliable UAV cooperative missions.

Whether it is formation maintenance or coordinated enclosing, traditional time triggered control strategies typically require periodic state exchange and control updates between unmanned aerial vehicles [19]. As the formation expands, this continuous communication mode will generate significant communication overhead, posing a severe challenge to the limited communication bandwidth, computing resources, and onboard energy of UAV platforms [20]. Especially in complex task environments, excessive consumption of communication resources may become a key bottleneck that restricts system scalability and task endurance. Event-triggered control, as an effective solution, provides a promising solution to alleviate the above problems by initiating communication and control updates only when the system state deviates from expected behavior beyond a specific threshold [21,22].

Nevertheless, most existing event-triggered strategies rely on fixed threshold mechanisms [23,24,25], which lack adaptability to dynamically evolving task conditions. Excessively conservative thresholds may sustain high communication rates, depleting resources, while overly relaxed thresholds risk degrading enclosing accuracy and formation stability. Therefore, designing an adaptive triggering mechanism that intelligently adjusts thresholds in response to real-time system dynamics is not merely a technical enhancement but an urgent necessity. Achieving an optimal balance between control performance and communication efficiency is essential for enabling scalable, resilient, and long-endurance UAV swarm operations.

This paper addresses the integrated the problem of controlling UAV formation target enclosing under limited communication resources. At its core is the proposal of a distributed cooperative control framework that combines “unified formation description, task decoupling architecture, and adaptive communication load reduction”. The principal contributions are as follows:

(1) A unified formation description method is developed based on geometric transformation parameter set. Abandoning the traditional static description based on relative position or orientation, the expected formation is defined as a composite geometric transformation of formation center translation, reference vector scaling, and rotation by introducing formation transformation operators. This method can uniformly and accurately describe the time-varying motion of the formation during travel, enclosing, and configuration switching, laying a mathematical model foundation for integrated control.
(2) A task-decoupled cooperative enclosing architecture is built. By designing virtual leaders and benchmarks with complementary functions, the complex enclosing task is decoupled into two sub-problems: global target tracking and local enclosing configuration generation. This architecture achieves the organic combination of formation control and target enclosing in a unified framework, supporting flexible transition of various formation patterns from equidistant enclosing to variable distance enclosing.
(3) A distributed event-triggering mechanism with internal dynamic variables is designed. To solve the problem of limited communication resources, an adaptive triggering condition based on local errors is designed, which dynamically manages the information exchange between UAVs by adjusting the communication threshold online through internal dynamic variables. Compared with static-triggering strategies, this mechanism can significantly reduce communication frequency and computational load while strictly ensuring system stability and enclosing accuracy.

## 2. Preparatory Knowledge and Problem Formulation

### 2.1. Model Establishment

This work investigates formation control and dynamic target enclosing for multi-UAV systems operating in a two-dimensional space at a fixed altitude. In a system consisting of n UAVs, assuming that the internal loop control is stable, each UAV is treated as a particle and described using the following kinematic model:

(1) $$\left\{\begin{array}{l} {\dot{p}}_{ix}\left(t\right)=\left\|v_{i}\left(t\right)\right\|\cos{\bar{\theta }}_{i}\left(t\right)\\ {\dot{p}}_{iy}\left(t\right)=\left\|v_{i}\left(t\right)\right\|\sin{\bar{\theta }}_{i}\left(t\right)\\ {\dot{v}}_{i}\left(t\right)=u_{i}\left(t\right)\\ {\dot{\bar{\theta }}}_{i}\left(t\right)=u_{i}^{\bar{\theta }}\left(t\right) \end{array}\right.,i\in \Theta$$

where $p_{i}\left(t\right)={\left(p_{ix}\left(t\right),\;p_{iy}\left(t\right)\right)}^{T}$, $v_{i}\left(t\right)={\left(v_{ix}\left(t\right),v_{iy}\left(t\right)\right)}^{T}$, and $u_{i}\left(t\right)={\left(u_{ix}\left(t\right),u_{iy}\left(t\right)\right)}^{T}$ are the position, velocity, and control input of the UAVs, respectively. $\Theta =\left\{1,2,\cdots ,n\right\}$ is the index set of the UAVs. ${\bar{\theta }}_{i}\left(t\right)$ is the heading angel of the UAVs, and $u_{i}^{\bar{\theta }}\left(t\right)$ is the control input of ${\bar{\theta }}_{i}\left(t\right)$.

To simplify controller design and focus on the cooperative strategy, the dynamic feedback linearization method is used to transform model (1) into a more manageable dual integrator model as the basic kinematic model for subsequent analysis:

(2) $$\left\{\begin{array}{l} {\dot{p}}_{i}\left(t\right)=v_{i}\left(t\right)\\ {\dot{v}}_{i}\left(t\right)=u_{i}\left(t\right) \end{array}\right.,i\in \Theta$$

To clearly describe the cooperative task of formation target enclosing and provide a unified reference framework, two virtual UAVs, ${\mathrm{UAV}}_{l}$ and ${\mathrm{UAV}}_{b}$, with specific functions, are introduced. The dynamic model of ${\mathrm{UAV}}_{l}$ is formulated as follows:

(3) $$\left\{\begin{array}{l} {\dot{p}}_{l}\left(t\right)=v_{l}\left(t\right)\\ {\dot{v}}_{l}\left(t\right)=u_{l}\left(t\right) \end{array}\right.$$

where $p_{l}\left(t\right)={\left(p_{lx}\left(t\right),p_{ly}\left(t\right)\right)}^{T}$ and $v_{l}\left(t\right)={\left(v_{lx}\left(t\right),v_{ly}\left(t\right)\right)}^{T}$ are the position and velocity of ${\mathrm{UAV}}_{l}$, respectively, and $u_{l}\left(t\right)$ is the control input of ${\mathrm{UAV}}_{l}$.

The dynamic model of ${\mathrm{UAV}}_{b}$ is formulated as follows:

(4) $$\left\{\begin{array}{l} {\dot{p}}_{b}\left(t\right)=v_{b}\left(t\right)\\ {\dot{v}}_{b}\left(t\right)=u_{b}\left(t\right) \end{array}\right.$$

where $p_{b}\left(t\right)$, $v_{b}\left(t\right)$, and $u_{b}\left(t\right)$ are the reference vector, reference variation, and reference control quantity of ${\mathrm{UAV}}_{b}$, respectively. It is noted that ${\mathrm{UAV}}_{b}$ is not an actual UAV. $u_{b}\left(t\right)$ represents the pre-order parameters; $p_{b}\left(t\right)$ and $v_{b}\left(t\right)$ are controlled by $u_{b}\left(t\right)$, and they are not considered as the actual position and velocity of ${\mathrm{UAV}}_{b}$.

### 2.2. Communication Topology

In multi-UAV cooperative control systems, information exchange is the foundation for collaboration. The complex communication relationships within the system are abstracted and modeled using graph theory. Undirected graph $G_{1}=\left(V_{1},\varepsilon_{1}\right)$ is defined. $V_{1}$ is the set of UAVs, with each node corresponding to an actual UAV. $\varepsilon_{1}$ is the communication set between UAVs. If $\left({\mathrm{UAV}}_{i},{\mathrm{UAV}}_{j}\right)\in \varepsilon_{1}$, ${\mathrm{UAV}}_{i}$ and ${\mathrm{UAV}}_{j}$ can communicate bidirectionally and are referred to as adjacent UAVs. The adjacent UAVs of ${\mathrm{UAV}}_{i}$ can be represented as $N_{i}$. The adjacency matrix $A_{1}=\left[a_{ij}\right]\in R^{n\times n}$ is defined to quantify the weights of communication connection, and satisfy the following:

(5) $$\left\{\begin{array}{l} a_{ij}>0,\left({\mathrm{UAV}}_{i},{\mathrm{UAV}}_{j}\right)\in \varepsilon_{1}\\ a_{ij}=0,\left({\mathrm{UAV}}_{i},{\mathrm{UAV}}_{j}\right)\notin \varepsilon_{1} \end{array}\right.$$

On this basis, the Laplacian matrix of graph $G_{1}$ is defined as $G_{1}$ $L=\left[l_{ij}\right]=D-A_{1}$, where $D=\mathrm{diag}\left(D_{1},D_{2},\cdots ,D_{n}\right)\in R^{n\times n}$ represents the in-degree matrix of $G_{1}$, $D_{i}=\sum_{j=1}^{n}a_{ij}$ is the weighted in-degree of ${\mathrm{UAV}}_{i}$.

The guidance relationship between actual UAVs and virtual UAVs is represented by a directed graph $G_{2}=\left(V_{2},\varepsilon_{2}\right)$, and $V_{2}$ includes all actual UAVs and virtual UAVs. $\varepsilon_{2}$ represents the communication relationship set between ${\mathrm{UAV}}_{i}$ and ${\mathrm{UAV}}_{\tilde{k}}$, $\tilde{k}=\left\{l,b\right\}$. When $\left({\mathrm{UAV}}_{i},{\mathrm{UAV}}_{\tilde{k}}\right)\in \varepsilon_{2}$, ${\mathrm{UAV}}_{i}$ can obtain the information from ${\mathrm{UAV}}_{\tilde{k}}$ and define the matrix $A_{2}=\left[a_{i\tilde{k}}\right]\in R^{n\times 2}$, and

(6) $$\left\{\begin{array}{l} a_{i\tilde{k}}>0,\left({\mathrm{UAV}}_{i},{\mathrm{UAV}}_{\tilde{k}}\right)\in \varepsilon_{2}\\ a_{i\tilde{k}}=0,\left({\mathrm{UAV}}_{i},{\mathrm{UAV}}_{\tilde{k}}\right)\notin \varepsilon_{2} \end{array}\right.$$

### 2.3. Problem Description

**Figure 1:**
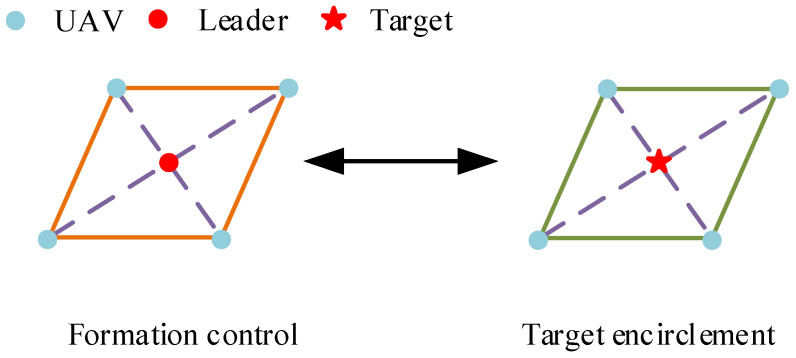
Schematic diagram of formation control and target enclosing.

The core of UAV formation target enclosing as an integrated cooperative task lies in the organic combination of two similar sub-tasks: formation control and target enclosing. However, current research usually studies the two issues separately as independent processes, neglecting the collaboration of multiple UAVs from the initial mission to the enclosing stage. As shown in Figure 1, formation control requires the UAVs swarm to form and maintain a specific spatial geometric configuration during movement. Target enclosing requires the UAV swarm to form a stable enclosing structure around the target. It usually needs to maintain a specific relative phase to achieve coordinated monitoring or attack. Although these two have different forms of expression, the former focuses on maintaining the formation in space, and the latter focuses on deploying and maintaining the formation around the target. However, their essence can be attributed to the problem of multiple UAVs moving in a coordinated manner around a dynamic center (formation center or target) in a specific pattern.

In the specific implementation of the enclosing strategy, existing methods are mostly limited to fixed distance enclosing, which requires all UAVs to maintain the same preset distance from the target, thus forming a fixed radius enclosing. Although this strategy simplifies control design, its drawbacks of fixed formation structure and insufficient flexibility are highlighted when dealing with complex terrain, obstacle avoidance, or performing specific tasks. This method can dynamically adjust the enclosing radius and formation geometry according to task requirements and environmental changes, achieving smooth transitions between various modes (such as from equidistant enclosing to variable distance enclosing), thereby significantly improving the adaptability of the enclosing strategy and task execution capability.

In addition, no matter in the creation of formation or target enclosing, continuous communication and control input updates are required between UAVs to maintain their formation. However, communication network bandwidth and computing resources are limited. How to reduce the pressure of communication between UAVs during target enclosing is an urgent problem to be solved. To reduce communication burden and frequent control updates in UAV teams, we introduce an intermittent communication protocol with a dynamic triggering feature.

## 3. Main Results

The following discussion will systematically explain the core control framework and its strategy. First, a new time-varying formation description method based on a geometric transformation parameter set is proposed, laying a theoretical foundation for cooperative enclosing tasks; second, an adaptive target enclosing model is constructed to achieve formation control and target enclosing in a unified framework; finally, this paper presents a dynamic event-triggered control strategy for resource-constrained formation target enclosing, complete with a rigorous analysis of its stability and proof of Zeno-free behavior.

### 3.1. Time-Varying Formation Description Based on Geometric Transformation Parameter Set

To build an integrated framework for multi-UAVs target enclosing and control, a primary and crucial foundation is as follows:

How to accurately and uniquely describe an expected UAV formation. Traditional formation description methods, such as those based on relative position or orientation, have inherent limitations in describing formations with basic geometric transformations such as rotation and scaling.

**Figure 2:**
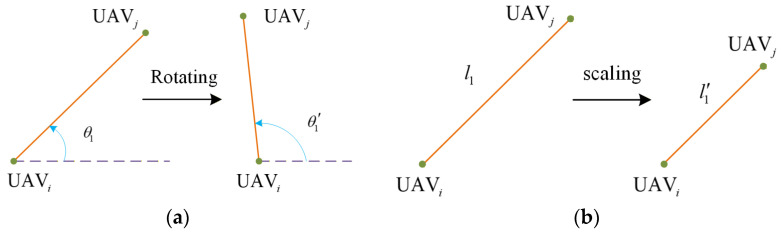
Schematic diagram of existing formation description methods. (**a**) orientation−based; (**b**) distance−based.

As shown in Figure 2a, based on the method of relative orientation, if there is no coordinated change in the relative orientation angle between UAVs after formation rotation, there will be a deviation between the actual formation and the expected formation. If the relative orientation between two UAVs is initially defined as $\theta_{1}$, the relative position between them is ${\theta_{1}}^{\prime }$ after rotational motion. If ${\theta_{1}}^{\prime }\neq \theta_{1}+2\pi$, the formation does not match the initially described formation.

In Figure 2b, the method using relative distance will also distort the formation after scaling if the distance is not adjusted according to the preset ratio. If the relative distance between UAVs is initially defined as $l_{1}$, the distance between them is ${l_{1}}^{\prime }$ after scaling motion. If ${l_{1}}^{\prime }\neq l_{1}$, the formation does not match the initially defined expected formation.

The core issue lies in the static nature of their formation definitions, overlooking the essential principles of geometric transformation for the entire swarm.

A formation description method is proposed based on the geometric transformation parameter set. The core idea of this method is to treat the expected formation of the entire UAV formation as a result generated by a unified translation, rotation, and scaling transformation applied to a standard reference vector. This descriptive method is compatible with the requirements of time-varying formations, and then the formation control and target enclosing can be achieved.

The geometric transformation parameter set of ${\mathrm{UAV}}_{i}$ is

(7) $$T_{i}\left(t\right)=\left\{p_{l}\left(t\right),p_{b}\left(t\right),d_{i}\left(t\right),X_{i}\left(t\right)\right\}$$

The entire formation can then be defined by the parameter set of all UAVs:

(8) $$\begin{array}{cc} T\left(n\right)= & \left\{T_{1}\left(t\right),T_{2}\left(t\right),\cdots ,T_{n}\left(t\right)\right\}\\ = & \left\{p_{l}\left(t\right),p_{b}\left(t\right),d_{1}\left(t\right),X_{1}\left(t\right),\cdots ,d_{n}\left(t\right),X_{n}\left(t\right)\right\} \end{array}$$

In the parameter set, $p_{l}\left(t\right)$ is the set center of the formation that defined by the position of virtual navigation ${\mathrm{UAV}}_{l}$. It determines the overall translational motion of the formation in space and serves as the reference origin of all UAV movements. $p_{b}\left(t\right)$ is the reference vector that is defined by ${\mathrm{UAV}}_{b}$. This vector is a directional reference on the unit circle, providing a unified reference frame for the rotation and scaling of the formation. $d_{i}\left(t\right)\geq 0$ is the scaling parameter of ${\mathrm{UAV}}_{i}$. It determines the radial distance of UAVs in the direction of the reference vector and controls the shape and size of the formation. $X_{i}\left(t\right)\in SO\left(2\right)$ is the rotation parameter. It is a two-dimensional unit orthogonal matrix that controls the rotation transformation of the UAV relative to the reference direction, and determines the phase of the UAV on the circumference.

Based on the above analysis, this parameter set includes three types of motion: rotation, scaling, and translation. The scaling motion is controlled by $d_{i}\left(t\right)$, the rotation motion is controlled by $X_{i}\left(t\right)$, and the translation motion depends on the change in the formation center $p_{l}\left(t\right)$. Both scaling and rotation motions are relative to ${\mathrm{UAV}}_{b}$, which is why ${\mathrm{UAV}}_{b}$ is used.

In three types of movements, the UAV formation mainly depends on the changes in $d_{i}\left(t\right)$ and $X_{i}\left(t\right)$, both of which take ${\mathrm{UAV}}_{b}$ as benchmarks. To simplify the operations in the subsequent controller design, the scaling and rotation parameters applied to the same object are merged. The formation transformation operator of ${\mathrm{UAV}}_{i}$ is

(9) $$C_{i}\left(t\right)=d_{i}\left(t\right)X_{i}\left(t\right),i\in \Theta$$

At this point, the geometric transformation parameter set can be equivalently represented as given by the following:

(10) $$T\left(n\right)=\left(p_{l}\left(t\right),p_{b}\left(t\right),C_{1}\left(t\right),\cdots ,C_{n}\left(t\right)\right)$$

**Remark** **1.** *The rotation matrix $X_{i}\left(t\right)$ preserves the orthogonality and orientation of the reference vector while $d_{i}\left(t\right)$ scales its magnitude. Their product $C_{i}\left(t\right)$ represents a simultaneous scaling and rotation transformation in the 2D plane. This is equivalent to applying $X_{i}\left(t\right)$ first (rotation) and then $d_{i}\left(t\right)$ (scaling), which is commutative in linear transformations under the assumption of isotropic scaling. In UAV formation control, scaling and rotation often occur simultaneously during maneuvers such as formation reshaping or target encircling. Combining them into a single operator simplifies the controller design by reducing the number of separate control variables. It also ensures that the geometric relationship between UAVs remains consistent during coordinated motions, which is essential for maintaining formation integrity under time-varying conditions.*

**Definition** **1.** *For the initial state of any UAV, if its final position satisfies*

(11) $$\underset{t\to \infty }{\lim}p_{i}\left(t\right)=p_{l}\left(t\right)+C_{i}\left(t\right)p_{b}\left(t\right),\;i\in \Theta$$

*then the control of the desired UAV formation is achieved by controlling parameter set $T\left(n\right)$.*

Definition 1 provides an intuitive representation of the expected formation: the expected position of each UAV is translated as a whole by the formation center $p_{l}\left(t\right)$, and then scaled and rotated by its unique formation transformation operator $C_{i}\left(t\right)$ on the reference vector $p_{b}\left(t\right)$ before being superimposed. This can enable more flexible formation switching of multiple UAVs, making the formation structure suitable for different mission scenarios.

**Figure 3:**
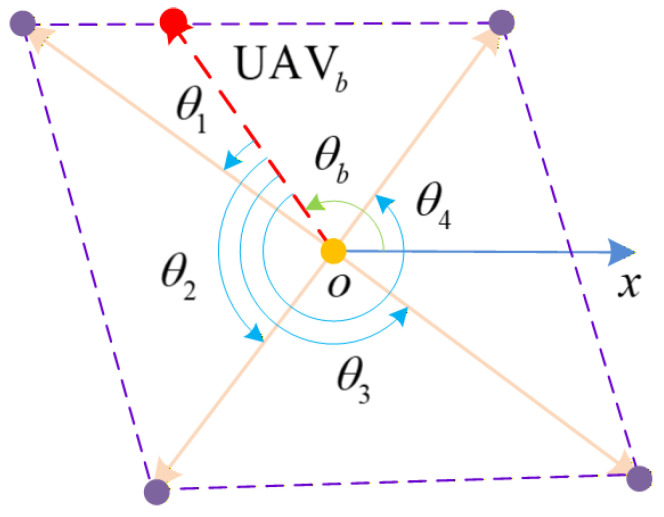
Schematic diagram of formation description.

To clarify the control meaning expressed in Definition 1 more clearly, the formation description diagram shown in Figure 3 is used for specific explanation. Consider a formation of UAVs is n=4, then

(12) $$p_{b}\left(t\right)={\left[\cos\left(\theta_{b}\left(t\right)\right)\;\;\;\;\sin\left(\theta_{b}\left(t\right)\right)\right]}^{T}$$

(13) $$d\left(t\right)=\left[d_{1}\left(t\right),d_{2}\left(t\right),d_{3}\left(t\right),d_{4}\left(t\right)\right],\;X_{i}\left(t\right)=\left(\begin{array}{cc} \cos\theta_{i}\left(t\right) & -\sin\theta_{i}\left(t\right)\\ \sin\theta_{i}\left(t\right) & \cos\theta_{i}\left(t\right) \end{array}\right)\;,i\in \Theta$$

$X_{i}\left(t\right)\in SO\left(2\right)$ meets the conditions. Considering $p_{b}\left(t\right)$ as constant, let $\theta_{b}\left(t\right)=\pi /2$, i.e., $p_{b}\left(t\right)={\left[0,1\right]}^{T}$; it can be seen that when $p_{b}\left(t\right)$ is determined, all UAVs in the formation will rotate counterclockwise along $p_{b}\left(t\right)$ with an angle $\theta_{i}\left(t\right)$ and then reach their respective positions. This indicates that the formation of UAV formations can be defined by Equation (10).

**Remark** **2.** *The formation description method in this section is oriented towards time-varying formations, which is mainly manifested in two aspects:*

*Benchmark dynamism: The benchmark vector $p_{b}\left(t\right)$ can be time-varying and can be achieved by controlling $\theta_{b}\left(t\right)$. This makes the formation perform rotations and other maneuvers as a rigid whole and maintains the formation unchanged. This is because all UAVs are used as benchmarks for formation maintenance.*

*Dynamic configuration: By independently and real-time controlling the formation transformation operator $C_{i}\left(t\right)$ of each UAV, i.e., independent control $d_{i}\left(t\right)$ and $X_{i}\left(t\right)$, it is possible to achieve the scaling of formation shape, rotation of formation orientation, and even continuous transformation of formation configuration (such as changing from rectangle to circle).*

**Remark** **3.** *Unlike affine transformation-based approaches, which typically define formations through linear mappings of a global reference frame, the proposed geometric transformation parameter set decouples translation, rotation, and scaling into interpretable and independently tunable parameters. This allows for real-time reconfiguration without recomputing the entire transformation matrix, thereby reducing computational overhead during rapid formation changes. In contrast to virtual structure methods that often rely on a rigid predefined geometry, our approach enables smooth transitions between diverse formation patterns (e.g., from circular to polygonal) by merely adjusting the scaling and rotation parameters of individual UAVs. Furthermore, compared to leader–follower frameworks where formation changes are propagated through hierarchical updates, our distributed parameter-based description supports parallel adjustment, enhancing responsiveness and scalability in dynamic environments.*

This description method transforms formation control into the control of a set of parameters with clear geometric meanings, providing convenience for subsequent integrated control design.

### 3.2. Design of Target Enclosing Model

This section constructs a dual leader formation enclosing model based on the formation description framework in Section 3.1. This model unifies the tasks of formation control and target enclosing that traditionally handled separately to be a control framework by assigning clear and complementary responsibilities to two virtual UAVs.

As a trajectory guidance UAV for a formation, the main function of ${\mathrm{UAV}}_{l}$ is to provide reference motion positions for the UAV formation. In the target enclosing mission, the task of trajectory guidance is to continuously bring UAVs closer to the target. Considering that the observation of the target state by airborne sensors is usually conducted at each sampling point, and taking into account this characteristic, a target enclosing model is established for ${\mathrm{UAV}}_{l}$. The motion rules of ${\mathrm{UAV}}_{l}$ approaching the target are as follows:

Step 1: Set the initial position $p_{l}\left(0\right)$ of ${\mathrm{UAV}}_{l}$ and define its initial velocity as $v_{l}\left(0\right)=e_{l}\left(0\right)\cdot \left\|v_{l}\right\|$, where $e_{l}\left(0\right)$ is the direction pointing towards the target at the initial moment.

Step 2: In each control cycle, based on the estimation of the target motion state at the current time, the motion direction of ${\mathrm{UAV}}_{l}$ at the current time can be determined as: $e_{l}\left(t\right)=\frac{p_{t}\left(t+\Delta t\right)-p_{l}\left(t\right)}{\left\|p_{t}\left(t+\Delta t\right)-p_{l}\left(t\right)\right\|}$, and $p_{t}\left(t+\Delta t\right)=p_{t}\left(t\right)+v_{t}\left(t\right)\cdot \Delta t$. The control input of ${\mathrm{UAV}}_{l}$ is $u_{l}\left(t\right)=\underset{\Delta t\to 0}{\lim}\frac{v_{l}\left(t\right)-v_{l}\left(t-\Delta t\right)}{\Delta t}$, where $p_{t}\left(t\right)$ and $v_{t}\left(t\right)$ are the position and velocity of the target at time t.

Step 3: Repeat Step 2 until the distance between ${\mathrm{UAV}}_{l}$ and the target meets the set error tolerance.

In the multi-UAV target enclosing, it is usually required that each UAV maintains the same distance from the target, and the phases of each UAV need to be coordinated. This imposes requirements on the formation transformation operator $C_{i}\left(t\right)$. The scaling parameters and rotation parameters are as follows:

(14) $$d_{i}\left(t\right)=d_{s}\left(t\right)/\left\|p_{b}\left(t\right)\right\|$$

(15) $$X_{i}\left(t\right)=\left(\begin{array}{cc} \cos\left(\theta_{i}\left(t\right)\right) & -\sin\left(\theta_{i}\left(t\right)\right)\\ \sin\left(\theta_{i}\left(t\right)\right) & \cos\left(\theta_{i}\left(t\right)\right) \end{array}\right),\;\theta_{i}\left(t\right)=\theta_{0}+\frac{2\pi \left(i-1\right)}{n}+\omega_{i}\left(t\right)$$

where $d_{s}\left(t\right)$ is the fixed distance radius of enclosing, which determines the size of the enclosing ring; $\theta_{0}\in \left[0,2\pi \right)$ is any phase angle initially set; the design of $\theta_{i}\left(t\right)$ ensures that the UAVs are evenly distributed in the enclosing ring; $\omega_{i}\left(t\right)$ is the rotational angular velocity of ${\mathrm{UAV}}_{i}$, which is designed for the situations where formation changes are required. In normal circumstances, it can be defined as $\omega_{i}=0$.

The core responsibility of ${\mathrm{UAV}}_{b}$ is to generate and maintain the expected enclosing formation around the target, with the reference vector designed as follows:

(16) $$p_{b}\left(t\right)={\left[\cos\left(\theta_{b}\left(t\right)\right)\;\sin\left(\theta_{b}\left(t\right)\right)\right]}^{T},\;\theta_{b}\left(t\right)=\omega t$$

where ω is the rotational angular velocity of ${\mathrm{UAV}}_{b}$, and it also denotes the angular velocity of the circular motion of UAVs. ω is switched according to different stages of the enclosing mission to enhance the flexibility of the strategy.

Define $\omega =\left\{\begin{array}{l} \omega_{1},\;\;\;tracking\;stage\\ \omega_{2},\;\;\;encirclement\;stage \end{array}\right.$; the setting of ω depends on the relative position between ${\mathrm{UAV}}_{l}$ and the target, that is, when $\left\|p_{t}-p_{l}\right\|$ is less than the set decision threshold, ${\mathrm{UAV}}_{l}$ catches up with the target, i.e., the formation is tracking the target.

In the above definition of ω, the turning angular velocity before and after encircling the target by the UAV formation is considered. This is mainly because before encircling the target, the velocity of the UAV formation depends on ${\mathrm{UAV}}_{l}$, while after encircling the target, the formation speed depends on the target velocity.

**Definition** **2.** *For any given initial state $p_{i}\left(0\right)$ and $v_{i}\left(0\right)$ of the UAV, if $\underset{t\to \infty }{\lim}p_{l}\left(t\right)=p_{t}\left(t\right)$ is satisfied while achieving the desired formation defined in Equation (11), it is said that the formation has been achieved by controlling the geometric transformation parameter set $T\left(n\right)$ to encircle the target in the desired formation.*

**Remark** **4.** *This model achieves an integrated design of formation control and target enclosing through a layered strategy. ${\mathrm{UAV}}_{l}$ is responsible for “target tracking” at the macro level, and ${\mathrm{UAV}}_{b}$ is responsible for “enclosing configuration” at the micro level. The actual UAV achieves collaboration by tracking the expected position determined by both of them and itself. This structure decomposes the complex formation target enclosing into two specific sub-problems, simplifying the analysis and design of the controller.*

### 3.3. Design of Target Enclosing Controller for UAV Formation Based on Event-Triggering Mechanism

**Figure 4:**
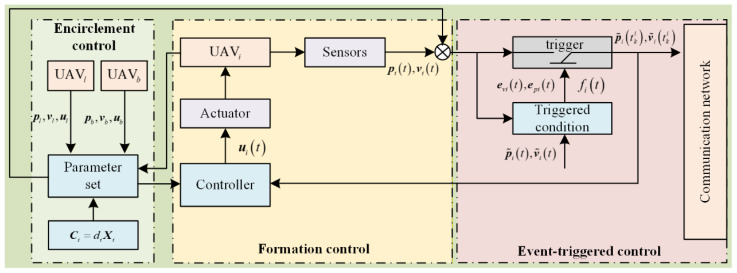
Schematic diagram of cooperative enclosing control framework.

The formation target control structure designed mainly includes three parts: formation control architecture, target enclosing control architecture, and event-triggered control architecture. The control schematic is shown in Figure 4.

The formation position error of ${\mathrm{UAV}}_{i}$ is

(17) $${\tilde{p}}_{i}\left(t\right)=p_{i}\left(t\right)-p_{l}\left(t\right)-C_{i}\left(t\right)p_{b}\left(t\right)$$

The formation speed error is

(18) $${\tilde{v}}_{i}\left(t\right)=v_{i}\left(t\right)-v_{l}\left(t\right)-C_{i}\left(t\right)v_{b}\left(t\right)-{\dot{C}}_{i}\left(t\right)p_{b}\left(t\right)$$

The above error measures the deviation between the actual and expected state defined by the virtual leader and formation transformation operator, and the control objective is to drive ${\tilde{p}}_{i}\left(t\right)$ and ${\tilde{v}}_{i}\left(t\right)$ approach 0.

**Definition** **3.** *For any ${\mathrm{UAV}}_{i}$ and ${\mathrm{UAV}}_{j}$, if there is a bounded time $t_{0}$ that when $t\geq t_{0}$, $\left\|{\tilde{v}}_{i}\left(t\right)-{\tilde{v}}_{j}\left(t\right)\right\|\to 0$ and $\left\|{\tilde{p}}_{i}\left(t\right)-{\tilde{p}}_{j}\left(t\right)\right\|\to 0$ are satisfied, and ${\tilde{p}}_{i}\left(t\right)\to 0$ and ${\tilde{v}}_{i}\left(t\right)\to 0$ are satisfied, then the expected formation of a multi-UAV formation is formed and maintained. If $\left\|p_{l}\left(t\right)-p_{t}\left(t\right)\right\|\to 0$ is satisfied at the same time, it is said that the UAV formation maintains the expected formation to encircle the target.*

In resource-constrained practical systems, it is often unrealistic to require continuous communication between UAVs to achieve precise collaboration. To address this, this section proposes an intermittent communication control strategy based on dynamic event-triggering. The core of this strategy is as follows: Only when the cumulative local state error of each UAV reaches a certain level, which is sufficient to trigger the communication conditions, will it exchange information with its neighbors and update the control instructions. In this way, the control performance is ensured to significantly save communication and compute resources.

The distributed dynamic event-triggered target enclosing control law for UAV formation is as follows:

(19) $$\left\{\begin{array}{c} u_{i}\left(t\right)=\vartheta_{i}\left(t\right)-\sum_{j=1}^{n}a_{ij}\left[k_{1}\left({\tilde{v}}_{i}\left(t_{k}^{i}\right)-{\tilde{v}}_{j}\left(t_{k}^{i}\right)\right)+k_{2}\left({\tilde{p}}_{i}\left(t_{k}^{i}\right)-{\tilde{p}}_{j}\left(t_{k}^{i}\right)\right)\right]\\ \;\;\;\;\;\;\;\;\;\;-k_{1}a_{ib}{\tilde{v}}_{i}\left(t\right)-k_{2}a_{ib}{\tilde{p}}_{i}\left(t\right)\\ \vartheta_{i}\left(t\right)={\ddot{C}}_{i}\left(t\right)p_{b}\left(t\right)+2{\dot{C}}_{i}\left(t\right)v_{b}\left(t\right)+C_{i}\left(t\right)u_{b}\left(t\right)+u_{l}\left(t\right) \end{array}\right.$$

where $\vartheta_{i}\left(t\right)$ is a feedforward compensation term used to counteract the expected dynamics caused by virtual leaders and formation transformations, thereby simplifying the analysis of closed-loop systems; $k_{1},k_{2}>0$ is the control gain. $t_{k}^{i}$ represents the k-th event-triggering time of ${\mathrm{UAV}}_{i}$. The UAV only communicates at the triggering time until the next triggering time $t_{k+1}^{i}$, during which there is no communication or exchange of status information. That is, within $t\in \left[t_{k}^{i},t_{k+1}^{i}\right)$, the UAV’s status remains unchanged from the value at $t_{k}^{i}$.

To facilitate the description and analysis of subsequent dynamic triggering strategies, the position and velocity measurement errors are as follows:

(20) $$e_{pi}\left(t\right)={\tilde{p}}_{i}\left(t_{k}^{i}\right)-{\tilde{p}}_{i}\left(t\right),t\in \left[t_{k}^{i},t_{k+1}^{i}\right)$$

(21) $$e_{vi}\left(t\right)={\tilde{v}}_{i}\left(t_{k}^{i}\right)-{\tilde{v}}_{i}\left(t\right),t\in \left[t_{k}^{i},t_{k+1}^{i}\right)$$

For any value of $t_{k}^{i},k=0,1,2,\cdots$, a dual-channel event-triggering function based on position and velocity is as follows:

(22) $$f_{i}\left(t\right)={\left\|e_{pi}\left(t\right)\right\|}^{2}+{\left\|e_{vi}\left(t\right)\right\|}^{2}-\Lambda_{1}{\left\|{\tilde{p}}_{i}\left(t\right)\right\|}^{2}-\Lambda_{2}{\left\|{\tilde{v}}_{i}\left(t\right)\right\|}^{2}\geq \eta_{i}\left(t\right)$$

(23) $$t_{k+1}^{i}=\inf\left\{t>t_{k}^{i}:f_{i}\left(t\right)\geq 0\right\}$$

where triggering parameters satisfy $0<\Lambda_{2}\leq \frac{2k_{1}a_{b}+k_{2}\lambda_{2}\left(L\right)-2k_{1}\sigma \left(n-1\right)-2k_{2}\sigma \left(n-1\right)}{2\bar{k}+2}$, $0<\Lambda_{1}\leq \frac{k_{2}\lambda_{2}\left(L\right)}{2\bar{k}+2}$, $0<\sigma \leq \frac{2k_{1}a_{b}+k_{2}\lambda_{2}\left(L\right)}{2\left(k_{1}+k_{2}\right)\left(n-1\right)}$, and $\bar{k}=\max\left\{\frac{k_{1}}{\sigma }\left(n-1\right)-1,\frac{k_{2}}{\sigma }\left(n-1\right)-1\right\}$. $\eta_{i}\left(t\right)$ is an internal dynamic variable, and the update rule is as follows:

(24) $${\dot{\eta }}_{i}\left(t\right)=-\beta \eta_{i}\left(t\right)+\left(\Lambda_{1}{\left\|{\tilde{p}}_{i}\left(t\right)\right\|}^{2}+\Lambda_{2}{\left\|{\tilde{v}}_{i}\left(t\right)\right\|}^{2}-{\left\|e_{pi}\left(t\right)\right\|}^{2}-{\left\|e_{vi}\left(t\right)\right\|}^{2}\right)$$

where $0<\bar{k}<\beta$, $\eta_{i}\left(0\right)>0$. At the same time, dynamic variables can be adjusted according to the actual situation to balance the trade-off between control accuracy and communication consumption.

According to the triggering conditions, it can be known that when ${\left\|e_{pi}\left(t\right)\right\|}^{2}+{\left\|e_{vi}\left(t\right)\right\|}^{2}-\Lambda_{1}{\left\|{\tilde{p}}_{i}\left(t\right)\right\|}^{2}-\Lambda_{2}{\left\|{\tilde{v}}_{i}\left(t\right)\right\|}^{2}\geq \eta_{i}\left(t\right)$, the event will be triggered. Then, within the time interval $t\in \left[t_{k}^{i},t_{k+1}^{i}\right)$, the system will update its state through a zero-order hold device and measure error $e_{pi}\left(t\right)$, and $e_{vi}\left(t\right)$ is reset to 0. Together with (24), this leads to

(25) $${\dot{\eta }}_{i}\left(t\right)\leq -\beta \eta_{i}\left(t\right)-\eta_{i}\left(t\right)=-\left(\beta +1\right)\eta_{i}\left(t\right)$$

where $\left(\beta +1\right)>0$; then

(26) $$\eta_{i}\left(t\right)>\eta_{i}\left(0\right)e^{-\left(\beta +1\right)t}>0$$

**Remark** **5.** *Compared with the static trigger (fixed threshold), the dynamic variable $\eta_{i}\left(t\right)$ introduces a time-varying trigger threshold. When the system error is large or the dynamics are severe, the positive term in (24) dominates and $\eta_{i}\left(t\right)$ increases, avoiding unnecessary frequent triggering. When the system approaches steady state, the $\eta_{i}\left(t\right)$ index decays, tightening the triggering conditions to ensure the final enclosing accuracy.*

**Remark** **6.** *This mechanism ensures that UAVs mostly remain in a communication silence at all times and only communicates and updates the control parameters “when necessary” (i.e., when the error accumulates to a certain level), significantly reducing the average communication frequency and computational load of the system.*

**Remark** **7.** *The control gains $k_{1},k_{2}$ are designed based on the Lyapunov stability analysis in Theorem 1, ensuring system convergence and Zeno-free behavior. The triggering parameters $\Lambda_{1},\Lambda_{2},\sigma ,\bar{k},\beta$ are tuned via simulations to achieve a trade-off between formation accuracy and communication frequency. Specifically, larger $\Lambda_{1},\Lambda_{2},\sigma ,\bar{k}$ reduce triggering times but may slightly increase steady-state error; β adjusts the dynamic threshold to suppress unnecessary triggers during transients. The values used in Section 4 reflect one feasible setup that balances performance and resource constraints.*

**Remark** **8.** *The initial value $\eta_{i}\left(0\right)$ can be tuned according to practical needs: smaller values favor faster initial convergence at the cost of more early-stage communication; larger values reduce initial communication but may slightly extend the transient period. In practice, $\eta_{i}\left(0\right)$ can be set based on the expected initial error magnitude or through offline tuning.*

### 3.4. System Stability and Zeno Free Behavior Analysis

**Theorem** **1.** *For a formation system composed of multiple UAVs, when the control gain and triggering parameters meet the above requirements, the controller (19) and the dynamic event-triggering mechanism (22) and (23) can enable the multi-UAV system to achieve the desired formation and encircle the target, while ensuring that no Zeno behavior occurs during the mission.*

**Proof of Theorem** **1.** Consider the following Lyapunov function:

(27) $$V\left(t\right)=\frac{1}{2}\sum_{i=1}^{n}k_{2}a_{ib}{\tilde{p}}_{i}^{T}\left(t\right){\tilde{p}}_{i}\left(t\right)+\frac{1}{2}\sum_{i=1}^{n}{\tilde{v}}_{i}^{T}\left(t\right){\tilde{v}}_{i}\left(t\right)+\sum_{i=1}^{n}\eta_{i}\left(t\right)$$

Derivate it, then

(28) $$\begin{array}{ll} \dot{V}\left(t\right) & =\sum_{i=1}^{n}k_{2}a_{ib}{\tilde{p}}_{i}^{T}\left(t\right){\dot{\tilde{p}}}_{i}\left(t\right)+\sum_{i=1}^{n}{\tilde{v}}_{i}^{T}\left(t\right){\dot{\tilde{v}}}_{i}\left(t\right)+\sum_{i=1}^{n}{\dot{\eta }}_{i}\left(t\right)\\ & =\sum_{i=1}^{n}k_{2}a_{ib}{\tilde{p}}_{i}^{T}\left(t\right){\tilde{v}}_{i}\left(t\right)+\sum_{i=1}^{n}{\tilde{v}}_{i}^{T}\left(t\right)\left[-\sum_{j=1}^{n}a_{ij}\left(k_{1}\left({\tilde{v}}_{i}\left(t_{k}^{i}\right)-{\tilde{v}}_{j}\left(t_{k}^{i}\right)\right)\right.\right.\\ & \left.+k_{2}\left({\tilde{p}}_{i}\left(t_{k}^{i}\right)-{\tilde{p}}_{j}\left(t_{k}^{i}\right)\right)-k_{1}a_{ib}{\tilde{v}}_{i}\left(t\right)-k_{2}a_{ib}{\tilde{p}}_{i}\left(t\right)\right]+\sum_{i=1}^{n}{\dot{\eta }}_{i}\left(t\right) \end{array}$$

then

(29) $$\begin{array}{ll} \dot{V}\left(t\right) & =-k_{1}\sum_{i=1}^{n}{\tilde{v}}_{i}^{T}\left(t\right)\sum_{j=1}^{n}a_{ij}\left(k_{1}\left({\tilde{v}}_{i}\left(t_{k}^{i}\right)-{\tilde{v}}_{j}\left(t_{k}^{i}\right)\right)\right.\\ & -k_{2}\sum_{i=1}^{n}{\tilde{v}}_{i}^{T}\left(t\right)\sum_{j=1}^{n}a_{ij}\left({\tilde{p}}_{i}\left(t_{k}^{i}\right)-{\tilde{p}}_{j}\left(t_{k}^{i}\right)\right)\\ & -k_{1}\sum_{i=1}^{n}a_{ib}{\tilde{v}}_{i}^{T}\left(t\right){\tilde{v}}_{i}\left(t\right)+\sum_{i=1}^{n}{\dot{\eta }}_{i}\left(t\right) \end{array}$$

Substituting (20), (21) and (24) into (29):

(30) $$\begin{array}{ll} \dot{V}\left(t\right) & =-k_{1}\sum_{i=1}^{n}{\tilde{v}}_{i}^{T}\left(t\right)\sum_{j=1}^{n}a_{ij}\left(k_{1}\left({\tilde{v}}_{i}\left(t\right)+e_{vi}\left(t\right)-{\tilde{v}}_{j}\left(t\right)-e_{vj}\left(t\right)\right)\right.\\ & -k_{2}\sum_{i=1}^{n}{\tilde{v}}_{i}^{T}\left(t\right)\sum_{j=1}^{n}a_{ij}\left({\tilde{p}}_{i}\left(t\right)+e_{pi}\left(t\right)-{\tilde{p}}_{j}\left(t\right)-e_{pj}\left(t\right)\right)\\ & -k_{1}\sum_{i=1}^{n}a_{ib}{\tilde{v}}_{i}^{T}\left(t\right){\tilde{v}}_{i}\left(t\right)-\beta \sum_{i=1}^{n}\eta_{i}\left(t\right)+\Lambda_{1}\sum_{i=1}^{n}{\left\|{\tilde{p}}_{i}\left(t\right)\right\|}^{2}\\ & +\Lambda_{2}\sum_{i=1}^{n}{\left\|{\tilde{v}}_{i}\left(t\right)\right\|}^{2}-\sum_{i=1}^{n}{\left\|e_{pi}\left(t\right)\right\|}^{2}-\sum_{i=1}^{n}{\left\|e_{vi}\left(t\right)\right\|}^{2} \end{array}$$

then

(31) $$\begin{array}{ll} \dot{V}\left(t\right) & =-k_{1}\sum_{i=1}^{n}{\tilde{v}}_{i}^{T}\left(t\right)L{\tilde{v}}_{i}\left(t\right)-k_{1}\sum_{i=1}^{n}n_{i}{\tilde{v}}_{i}^{T}\left(t\right)e_{vi}\left(t\right)+k_{1}\sum_{i=1}^{n}{\tilde{v}}_{i}^{T}\left(t\right)\sum_{j\in N_{i}}e_{vj}\left(t\right)\\ & -k_{2}\sum_{i=1}^{n}{\tilde{v}}_{i}^{T}\left(t\right)L{\tilde{p}}_{i}\left(t\right)-k_{2}\sum_{i=1}^{n}n_{i}{\tilde{v}}_{i}^{T}\left(t\right)e_{pi}\left(t\right)+k_{2}\sum_{i=1}^{n}{\tilde{v}}_{i}^{T}\left(t\right)\sum_{j\in N_{i}}e_{pj}\left(t\right)\\ & -k_{1}\sum_{i=1}^{n}a_{ib}{\tilde{v}}_{i}^{T}\left(t\right){\tilde{v}}_{i}\left(t\right)-\beta \sum_{i=1}^{n}\eta_{i}\left(t\right)+\Lambda_{1}\sum_{i=1}^{n}{\left\|{\tilde{p}}_{i}\left(t\right)\right\|}^{2}\\ & +\Lambda_{2}\sum_{i=1}^{n}{\left\|{\tilde{v}}_{i}\left(t\right)\right\|}^{2}-\sum_{i=1}^{n}{\left\|e_{pi}\left(t\right)\right\|}^{2}-\sum_{i=1}^{n}{\left\|e_{vi}\left(t\right)\right\|}^{2} \end{array}$$

where $n_{i}$ represent the number of adjacent UAVs for the i-th UAV at time t, satisfying $n_{i}\leq n-1$. Due to $\sum_{i=1}^{n}\sum_{j\in N_{i}}e_{vj}\left(t\right)=\sum_{j\in N_{i}}n_{i}e_{vj}\left(t\right)$, and $\sum_{i=1}^{n}\sum_{j\in N_{i}}e_{pj}\left(t\right)=\sum_{j\in N_{i}}n_{i}e_{pj}\left(t\right)$, according to the Young inequation, it is obtained that

(32) $$-k_{1}\sum_{i=1}^{n}n_{i}{\tilde{v}}_{i}^{T}\left(t\right)e_{vi}\left(t\right)\leq \frac{k_{1}\sigma }{2}\sum_{i=1}^{n}\left(n-1\right){\left\|{\tilde{v}}_{i}\left(t\right)\right\|}^{2}+\frac{k_{1}}{2\sigma }\sum_{i=1}^{n}\left(n-1\right){\left\|e_{vi}\left(t\right)\right\|}^{2}$$

(33) $$\begin{array}{ll} k_{1}\sum_{i=1}^{n}{\tilde{v}}_{i}^{T}\left(t\right)\sum_{j\in N_{i}}e_{vj}\left(t\right) & \leq \frac{k_{1}\sigma }{2}\sum_{i=1}^{n}\left(n-1\right){\left\|{\tilde{v}}_{i}\left(t\right)\right\|}^{2}+\frac{k_{1}}{2\sigma }\sum_{i=1}^{n}\sum_{j\in N_{i}}e_{vj}^{2}\left(t\right)\\ & \leq \frac{k_{1}\sigma }{2}\sum_{i=1}^{n}\left(n-1\right){\left\|{\tilde{v}}_{i}\left(t\right)\right\|}^{2}+\frac{k_{1}}{2\sigma }\sum_{i=1}^{n}\left(n-1\right){\left\|e_{vi}\left(t\right)\right\|}^{2} \end{array}$$

(34) $$-k_{2}\sum_{i=1}^{n}{\tilde{v}}_{i}^{T}\left(t\right)L{\tilde{p}}_{i}\left(t\right)\leq -\left(\frac{k_{2}\lambda \left(L\right)}{2}\sum_{i=1}^{n}{\left\|{\tilde{v}}_{i}\left(t\right)\right\|}^{2}+\frac{k_{2}\lambda \left(L\right)}{2}\sum_{i=1}^{n}{\left\|{\tilde{p}}_{i}\left(t\right)\right\|}^{2}\right)$$

(35) $$-k_{2}\sum_{i=1}^{n}n_{i}{\tilde{v}}_{i}^{T}\left(t\right)e_{pi}\left(t\right)\leq \frac{k_{2}\sigma }{2}\sum_{i=1}^{n}\left(n-1\right){\left\|{\tilde{v}}_{i}\left(t\right)\right\|}^{2}+\frac{k_{2}}{2\sigma }\sum_{i=1}^{n}\left(n-1\right){\left\|e_{pi}\left(t\right)\right\|}^{2}$$

(36) $$\begin{array}{ll} k_{2}\sum_{i=1}^{N}{\tilde{v}}_{i}^{T}\left(t\right)\sum_{j\in N_{i}}e_{pj}\left(t\right) & \leq \frac{k_{2}\sigma }{2}\sum_{i=1}^{n}\left(n-1\right){\left\|{\tilde{v}}_{i}\left(t\right)\right\|}^{2}+\frac{k_{2}}{2\sigma }\sum_{i=1}^{n}\sum_{j\in N_{i}}e_{pj}^{2}\left(t\right)\\ & \leq \frac{k_{2}\sigma }{2}\sum_{i=1}^{n}\left(n-1\right){\left\|{\tilde{v}}_{i}\left(t\right)\right\|}^{2}+\frac{k_{2}}{2\sigma }\sum_{i=1}^{n}\left(n-1\right){\left\|e_{pi}\left(t\right)\right\|}^{2} \end{array}$$

Substituting (32)–(36) into (31), then

(37) $$\begin{array}{ll} \dot{V}\left(t\right) & \leq -k_{1}a_{b}\sum_{i=1}^{n}{\left\|{\tilde{v}}_{i}\left(t\right)\right\|}^{2}+k_{1}\sigma \sum_{i=1}^{n}\left(n-1\right){\left\|{\tilde{v}}_{i}\left(t\right)\right\|}^{2}+\frac{k_{1}}{\sigma }\sum_{i=1}^{n}\left(n-1\right){\left\|e_{vi}\left(t\right)\right\|}^{2}\\ & -\frac{k_{2}\lambda \left(L\right)}{2}\sum_{i=1}^{n}{\left\|{\tilde{v}}_{i}\left(t\right)\right\|}^{2}-\frac{k_{2}\lambda \left(L\right)}{2}\sum_{i=1}^{n}{\left\|{\tilde{p}}_{i}\left(t\right)\right\|}^{2}\\ & +k_{2}\sigma \sum_{i=1}^{n}\left(n-1\right){\left\|{\tilde{v}}_{i}\left(t\right)\right\|}^{2}+\frac{k_{2}}{\sigma }\sum_{i=1}^{n}\left(n-1\right){\left\|e_{pi}\left(t\right)\right\|}^{2}\\ & -\beta \sum_{i=1}^{n}\eta_{i}\left(t\right)+\Lambda_{1}\sum_{i=1}^{n}{\left\|{\tilde{p}}_{i}\left(t\right)\right\|}^{2}+\Lambda_{2}\sum_{i=1}^{n}{\left\|{\tilde{v}}_{i}\left(t\right)\right\|}^{2}\\ & -\sum_{i=1}^{n}{\left\|e_{pi}\left(t\right)\right\|}^{2}-\sum_{i=1}^{n}{\left\|e_{vi}\left(t\right)\right\|}^{2} \end{array}$$

and then

(38) $$\begin{array}{ll} \dot{V}\left(t\right) & \leq \left(-k_{1}a_{b}+k_{1}\sigma \left(n-1\right)-\frac{k_{2}\lambda \left(L\right)}{2}+k_{2}\sigma \left(n-1\right)+\Lambda_{2}\right)\sum_{i=1}^{n}{\left\|{\tilde{v}}_{i}\left(t\right)\right\|}^{2}\\ & +\left(\frac{k_{1}}{\sigma }\left(n-1\right)-1\right)\sum_{i=1}^{n}{\left\|e_{vi}\left(t\right)\right\|}^{2}+\left(\Lambda_{1}-\frac{k_{2}\lambda \left(L\right)}{2}\right)\sum_{i=1}^{n}{\left\|{\tilde{p}}_{i}\left(t\right)\right\|}^{2}\\ & +\left(\frac{k_{2}}{\sigma }\left(n-1\right)-1\right)\sum_{i=1}^{n}{\left\|e_{pi}\left(t\right)\right\|}^{2}-\beta \sum_{i=1}^{n}\eta_{i}\left(t\right) \end{array}$$

According to the triggering conditions:

(39) $$\sum_{i=1}^{n}{\left\|e_{pi}\left(t\right)\right\|}^{2}+\sum_{i=1}^{n}{\left\|e_{vi}\left(t\right)\right\|}^{2}\leq \;\Lambda_{1}\sum_{i=1}^{n}{\left\|{\tilde{p}}_{i}\left(t\right)\right\|}^{2}+\Lambda_{2}\sum_{i=1}^{n}{\left\|{\tilde{v}}_{i}\left(t\right)\right\|}^{2}+\sum_{i=1}^{n}\eta_{i}\left(t\right)$$

Take $\bar{k}=\max\left\{\frac{k_{1}}{\sigma }\left(n-1\right)-1,\frac{k_{2}}{\sigma }\left(n-1\right)-1\right\}$, substituting (39) into (38); then

(40) $$\begin{array}{ll} \dot{V}\left(t\right) & \leq \left(-k_{1}a_{b}+k_{1}\sigma \left(n-1\right)-\frac{k_{2}\lambda \left(L\right)}{2}+k_{2}\sigma \left(n-1\right)+\Lambda_{2}\left(\bar{k}+1\right)\right)\sum_{i=1}^{n}{\left\|{\tilde{v}}_{i}\left(t\right)\right\|}^{2}\\ & +\left(\Lambda_{1}\left(\bar{k}+1\right)-\frac{k_{2}\lambda \left(L\right)}{2}\right)\sum_{i=1}^{n}{\left\|{\tilde{p}}_{i}\left(t\right)\right\|}^{2}+\left(\bar{k}-\beta \right)\sum_{i=1}^{n}\eta_{i}\left(t\right) \end{array}$$

When σ, $\Lambda_{1}$, $\Lambda_{2}$, and β meet the design objectives, it can be inferred that $\dot{V}\left(t\right)\leq 0$. This indicates that UAV formations can achieve the desired formation to encircle targets.

The following demonstrates that no Zeno behavior occurs in the system throughout the entire enclosing process.

When the triggering condition (23) is met, $\left(k+1\right)\mathrm{th}$ is activated, and then

(41) $$\underset{t\to t_{k+1}^{i-}}{\lim}\left({\left\|e_{pi}\left(t\right)\right\|}^{2}+{\left\|e_{vi}\left(t\right)\right\|}^{2}\right)=\Lambda_{1}{\left\|{\tilde{p}}_{i}\left(t\right)\right\|}^{2}+\Lambda_{2}{\left\|{\tilde{v}}_{i}\left(t\right)\right\|}^{2}+\eta_{i}\left(t\right)$$

Taking the Dini derivative on the left side of the equation, it is obtained that

(42) $$\begin{array}{ll} D^{+}\left({\left\|e_{pi}\left(t\right)\right\|}^{2}+{\left\|e_{vi}\left(t\right)\right\|}^{2}\right) & =\frac{d}{dt}e_{pi}^{T}\left(t\right)e_{pi}\left(t\right)+\frac{d}{dt}e_{vi}^{T}\left(t\right)e_{vi}\left(t\right)\\ & =2e_{pi}^{T}\left(t\right)e_{vi}\left(t\right)+2e_{vi}^{T}\left(t\right){\dot{e}}_{vi}\left(t\right)\\ & \leq 2\left\|e_{pi}\left(t\right)\right\|\left\|e_{vi}\left(t\right)\right\|+2\left\|e_{vi}\left(t\right)\right\|\left\|{\dot{e}}_{vi}\left(t\right)\right\|\\ & =2\left\|e_{vi}\left(t\right)\right\|\left(\left\|e_{pi}\left(t\right)\right\|+\left\|{\dot{e}}_{vi}\left(t\right)\right\|\right) \end{array}$$

According to the definitions of $e_{pi}\left(t\right)$ and $e_{vi}\left(t\right)$, it is obtained that

(43) $$\left\{\begin{array}{l} \left\|{\dot{e}}_{pi}\left(t\right)\right\|=\left\|e_{vi}\left(t\right)\right\|\\ \begin{array}{l} \left\|{\dot{e}}_{vi}\left(t\right)\right\|=\left\|-{\dot{\tilde{v}}}_{i}\left(t\right)\right\|=\left\|\left.k_{1}a_{ib}{\tilde{v}}_{i}\left(t\right)+k_{2}a_{ib}{\tilde{p}}_{i}\left(t\right)\right\|\right.\\ \;\;\;\;\;\;\;\;\;\;\;\;\;\;+\sum_{j=1}^{n}a_{ij}\left[k_{1}\left({\tilde{v}}_{i}\left(t_{k}^{i}\right)-{\tilde{v}}_{j}\left(t_{k}^{i}\right)\right)+k_{2}\left({\tilde{p}}_{i}\left(t_{k}^{i}\right)-{\tilde{p}}_{j}\left(t_{k}^{i}\right)\right)\right] \end{array} \end{array}\right.$$

Let $\Delta_{i}$, $\mu_{i}$, and $\delta_{i}$, respectively, denote the maximum values of $\left\|e_{vi}\left(t\right)\right\|$, $\left\|{\dot{e}}_{vi}\left(t\right)\right\|$ and $\left\|e_{pi}\left(t\right)\right\|$ at time interval $t\in \left[t_{k}^{i},t_{k+1}^{i}\right)$; that is, $\Delta_{i}=\underset{t\in \left[t_{k}^{i},t_{k+1}^{i}\right)}{\max}\left\|e_{vi}\left(t\right)\right\|$, $\mu_{i}=\underset{t\in \left[t_{k}^{i},t_{k+1}^{i}\right)}{\max}\left\|{\dot{e}}_{vi}\left(t\right)\right\|$, and $\delta_{i}=\underset{t\in \left[t_{k}^{i},t_{k+1}^{i}\right)}{\max}\left\|e_{pi}\left(t\right)\right\|$.

Then

(44) $$D^{+}\left({\left\|e_{pi}\left(t\right)\right\|}^{2}+{\left\|e_{vi}\left(t\right)\right\|}^{2}\right)\leq {\left\|e_{pi}\left(t\right)\right\|}^{2}+{\left\|e_{vi}\left(t\right)\right\|}^{2}+2\Delta_{i}\left(\mu_{i}+\delta_{i}\right)$$

Due to $e_{pi}\left(t_{k}^{i}\right)=0$, $e_{vi}\left(t_{k}^{i}\right)=0$, then the inequality is as follows:

(45) $${\left\|e_{pi}\left(t\right)\right\|}^{2}+{\left\|e_{vi}\left(t\right)\right\|}^{2}\leq 2\Delta_{i}\left(\mu_{i}+\delta_{i}\right)\left(e^{t-t_{k}^{i}}-1\right)$$

When $t\to t_{k+1}^{i-}$,

(46) $$t_{k+1}^{i-}-t_{k}^{i}\geq \ln\left(\frac{1}{2\Delta_{i}\left(\mu_{i}+\delta_{i}\right)}\left(\Lambda_{1}{\left\|{\tilde{p}}_{i}\left(t\right)\right\|}^{2}+\Lambda_{2}{\left\|{\tilde{v}}_{i}\left(t\right)\right\|}^{2}+\eta_{i}\left(t\right)\right)+1\right)$$

When ${\tilde{p}}_{i}\left(t\right)\neq 0$ or ${\tilde{v}}_{i}\left(t\right)\neq 0$, $t_{k+1}^{i-}-t_{k}^{i}>0$; when ${\tilde{p}}_{i}\left(t\right)=0$ and ${\tilde{v}}_{i}\left(t\right)=0$, due to $\eta_{i}\left(t\right)>0$, $t_{k+1}^{i-}-t_{k}^{i}>0$ is obtained. It can be inferred that there is a strict lower bound greater than 0 for any triggering time interval, indicating there is no Zeno behavior. □

Throughout the stability analysis, we assume that the communication graph remains connected in the sense that all UAVs are able to communicate when triggered. Although the event-triggered mechanism reduces the frequency of data exchange, it does not alter the underlying graph connectivity, and thus the spectral properties of the Laplacian matrix used in the proof remain valid.

## 4. Simulation Verification

The simulation results validate the efficacy of the control strategy across four different test cases. In Scenario I, by changing the formation parameters, the control performance of multi-UAVs in a time-varying formation to encircle uniform targets is verified; in Scenario II, the feasibility using equidistant rotating formations to encircle uniform targets is verified under communication delays; in Scenario III, the feasibility of the variable-pitch rotational formation for encircling a maneuvering target is verified. Finally, comparative simulations are conducted, verifying the substantial communication resource savings achieved by the proposed dynamic triggering mechanism.

We have made modifications to all the relevant symbols in the figures and tables.

### 4.1. Time-Varying Formation to Encircle Uniform Target

Set the control gain is $k_{1}=0.3,k_{2}=0.2$, the triggering parameters β=0.1, $\Lambda_{1}=\Lambda_{2}=0.01$, and σ=0.05. The rotational angular velocity is ω=0; the initial phase angle is $\left[\frac{5\pi }{4},\frac{3\pi }{4},\frac{7\pi }{4},\frac{\pi }{4}\right]$; the phase angle within 30 s is $\left[\frac{\pi }{6},\frac{5\pi }{6},\frac{7\pi }{6},-\frac{\pi }{6}\right]$, and the enclosing radius is $d_{i}=40$; the phase angle within 31–50 s is $\left[\frac{\pi }{4},\frac{3\pi }{4},\frac{5\pi }{4},-\frac{\pi }{4}\right]$, and the enclosing radius is $d_{i}=30$; the phase angle within 51–60 s is $\left[\frac{\pi }{4},\frac{5\pi }{8},\frac{5\pi }{4},-\frac{\pi }{8}\right]$, and the enclosing radius is $d_{i}=20$.

**Figure 5:**
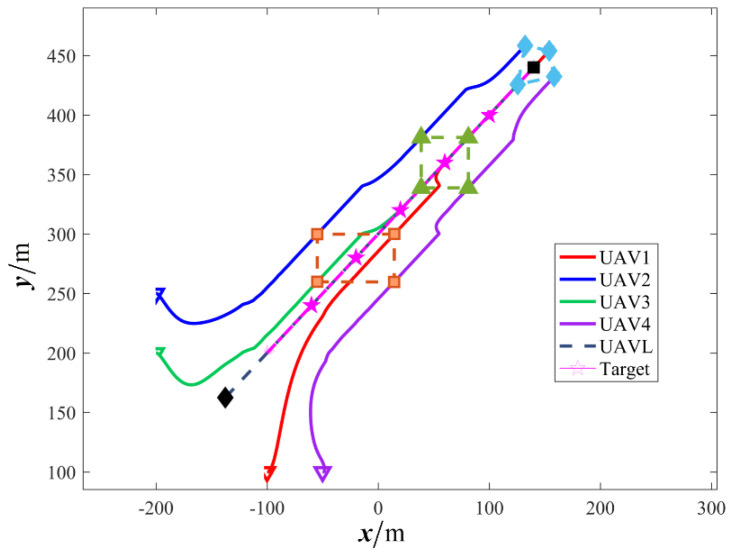
Scenario I: time−varying formation encircling uniform target trajectory.

**Figure 6:**
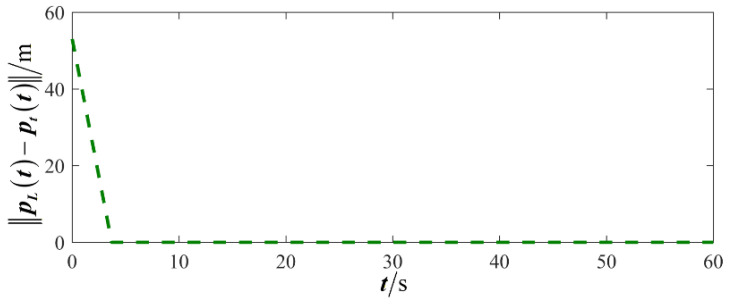
Scenario I: relative distance between virtual leader and target.

**Figure 7:**
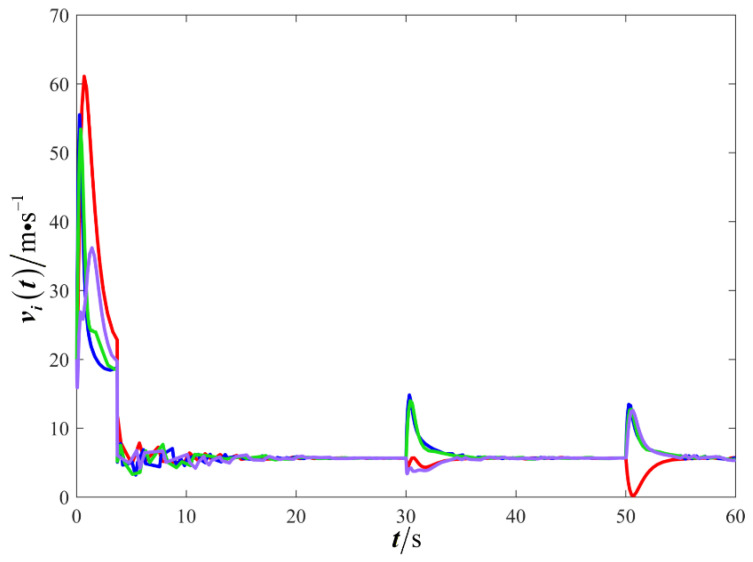
Scenario I: changes in UAV velocity.

**Figure 8:**
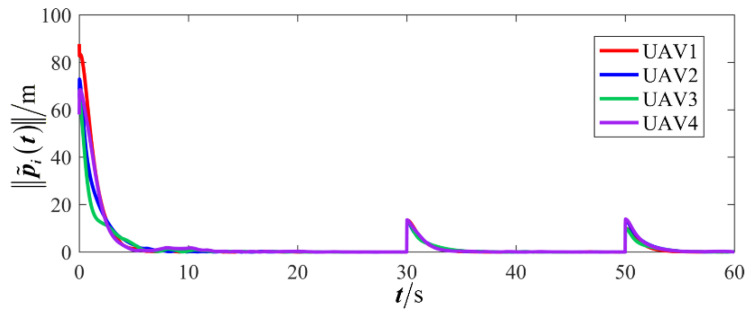
Scenario I: formation position error.

**Figure 9:**
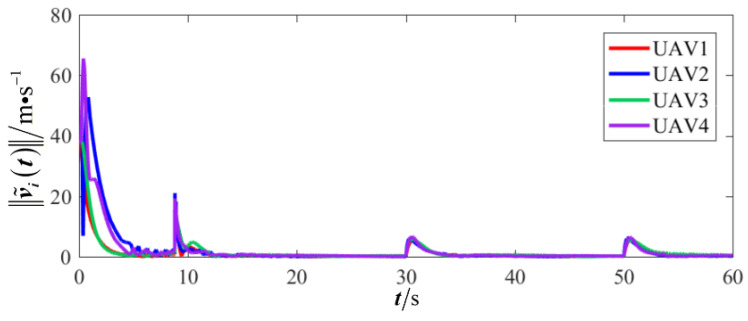
Scenario I: formation velocity error.

**Figure 10:**
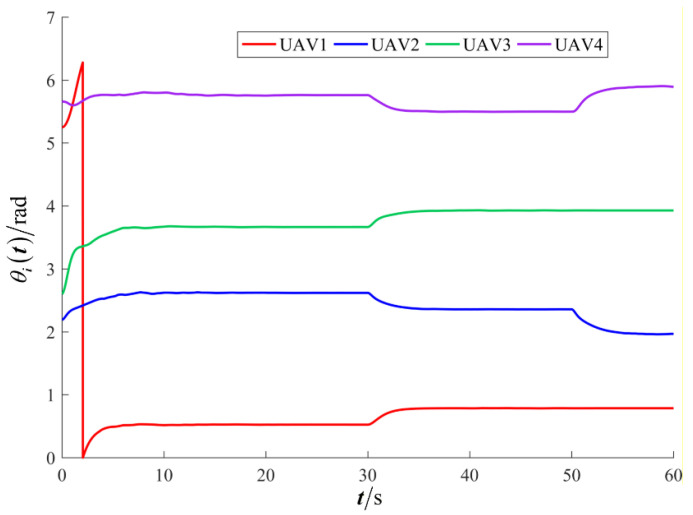
Scenario I: the phase of the UAV relative to the target.

**Figure 11:**
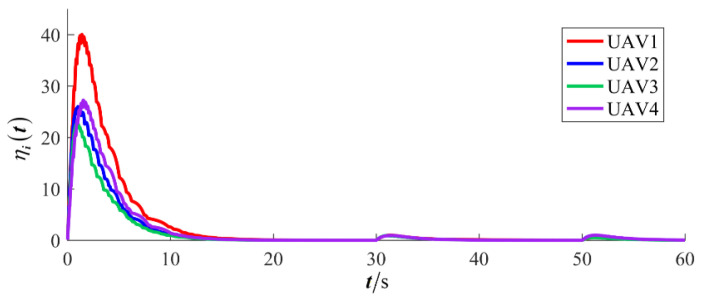
Scenario I: dynamic variable.

**Figure 12:**
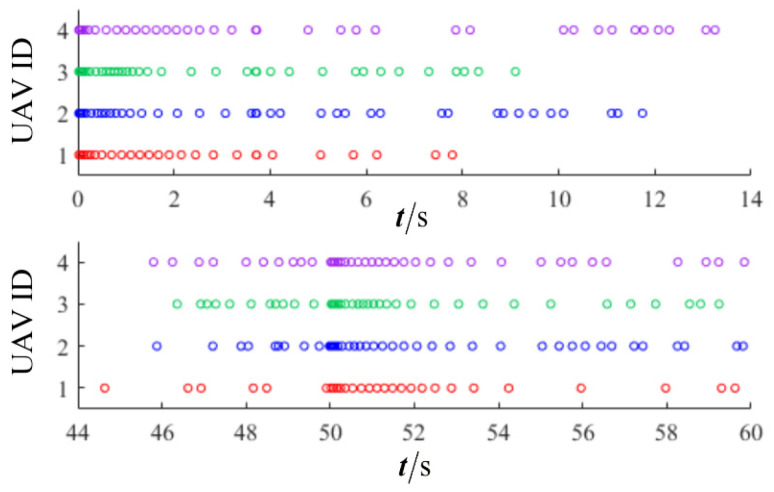
Scenario I: trigger distribution.

**Figure 13:**
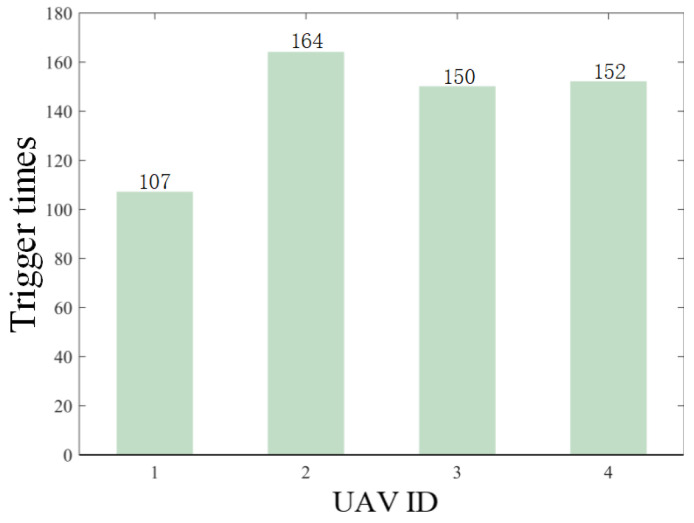
Scenario I: trigger times.

The results are presented in Figure 5, Figure 6, Figure 7, Figure 8, Figure 9, Figure 10, Figure 11, Figure 12 and Figure 13. Figure 5 depicts the trajectory evolution process of UAVs in encircling uniformly moving targets under time-varying formation control. It can be seen that in the initial stage, the UAVs quickly assemble into a rectangular formation and approach the target; then, under the control of preset formation parameters, the formation gradually transitions from a rectangle to a square and evolves into a diamond-like configuration. At the same time, the formation transformation and the target enclosing mission are successfully completed. Figure 6 shows the variation in the relative distance between virtual leader and target over time. This distance gradually converges to zero, indicating that the virtual leader has achieved asymptotic tracking of the target. Figure 7 illustrates the dynamic process of the speed changes in each UAV, indicating that under the influence of the control strategy; the speeds of all UAVs tend to be consistent and eventually remain stable, demonstrating excellent coordination consistency. Figure 8 and Figure 9, respectively, show the evolution process of formation position and velocity error. These two errors gradually converge over time. In particular, after the formation transition is completed, although there is a brief fluctuation, it quickly decays to zero, verifying the effectiveness of the designed control law in adjusting formation deviation and ensuring formation stability. Figure 10 shows the phase distribution of each UAV relative to the target, indicating that the UAVs maintain good phase coordination during the enclosing process, forming a uniformly distributed enclosing ring. Figure 11 shows the trend of changes in dynamic variables. Table 1 shows the system convergence times under different initial value $\eta_{i}\left(0\right)$. The trigger distribution diagram in Figure 12 shows that the triggering events are mainly concentrated in the initial adjustment stage, and then the triggering interval gradually increases. This indicates that the communication burden of the system is significantly reduced in the stable stage. Figure 13 shows the number of event-triggering of each UAV in the scene.

### 4.2. Isometric Rotating Formation to Encircle Uniform Velocity Target

Let the control gain and trigger parameters be unchanged; the enclosing radius is $d_{i}=50$, the rotational angular velocity is ω=0.4, the initial phase angle is $\left[\pi ,\frac{\pi }{2},\frac{3\pi }{2},0\right]$, and subsequent phase angle updates satisfy (16). To evaluate robustness under non-ideal communication, a bounded time-varying delay of $\left[0,50\right]\;\mathrm{ms}$ is introduced in the inter-UAV data exchange.

**Figure 14:**
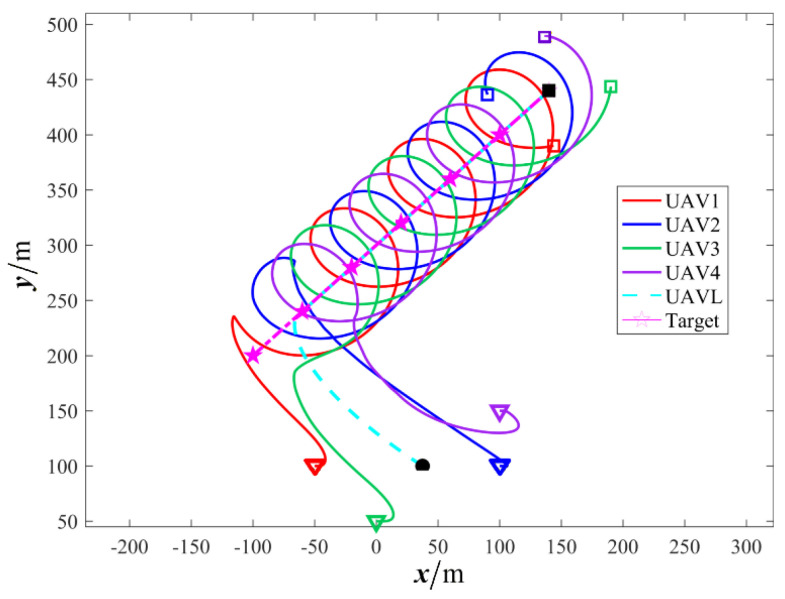
Scenario II: fixed−distance UAV formation trajectory for encircling a constant−velocity target.

**Figure 15:**
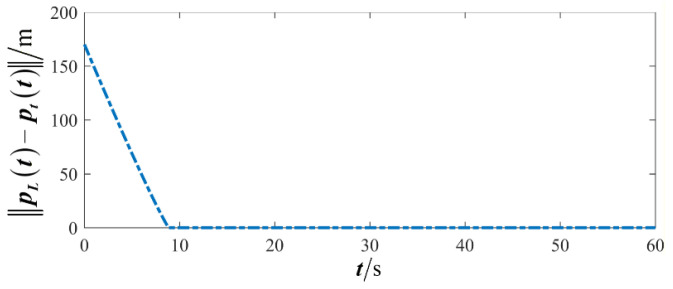
Scenario II: relative distance between virtual leader and target.

**Figure 16:**
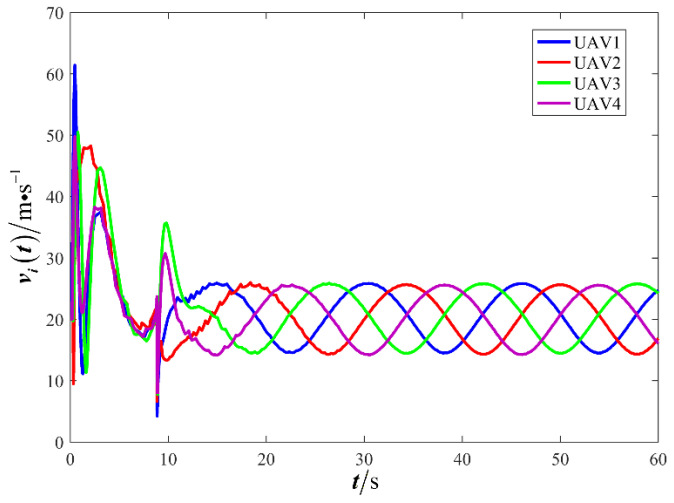
Scenario II: changes in UAV velocity.

**Figure 17:**
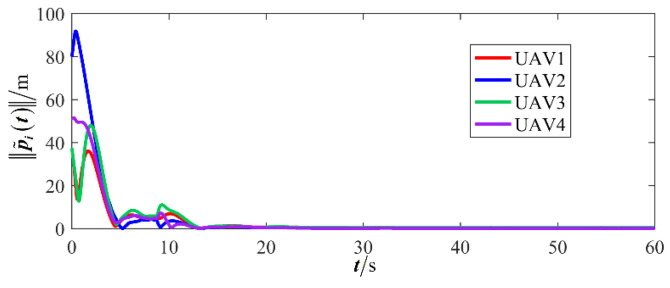
Scenario II: formation position error.

**Figure 18:**
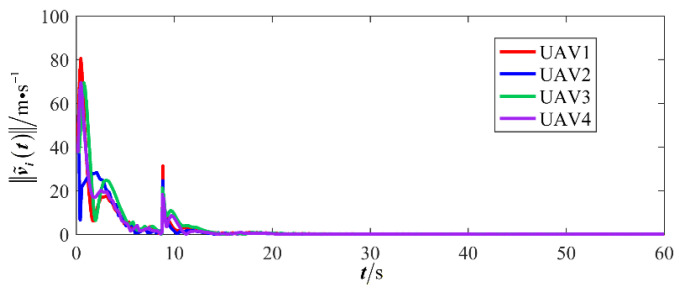
Scenario II: formation velocity error.

**Figure 19:**
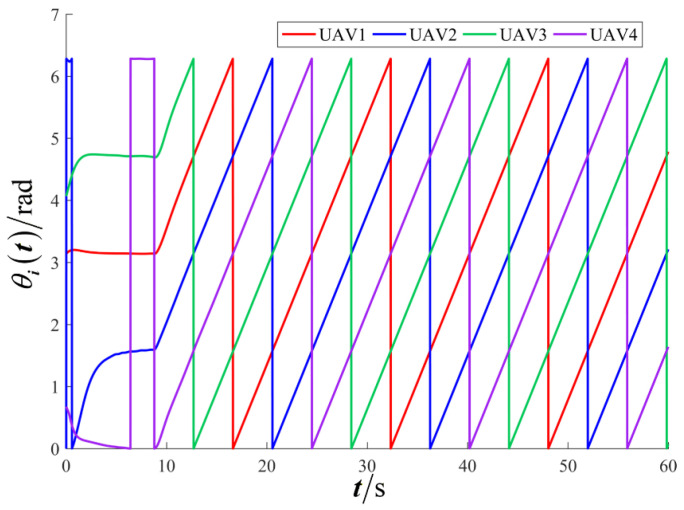
Scenario II: phase of the UAVs relative to the target.

**Figure 20:**
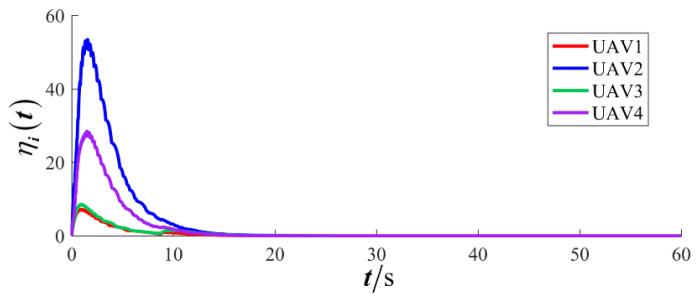
Scenario II: dynamic variable.

**Figure 21:**
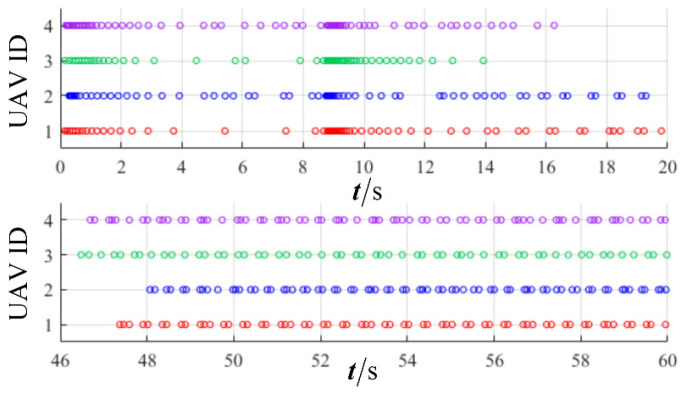
Scenario II: trigger distribution.

**Figure 22:**
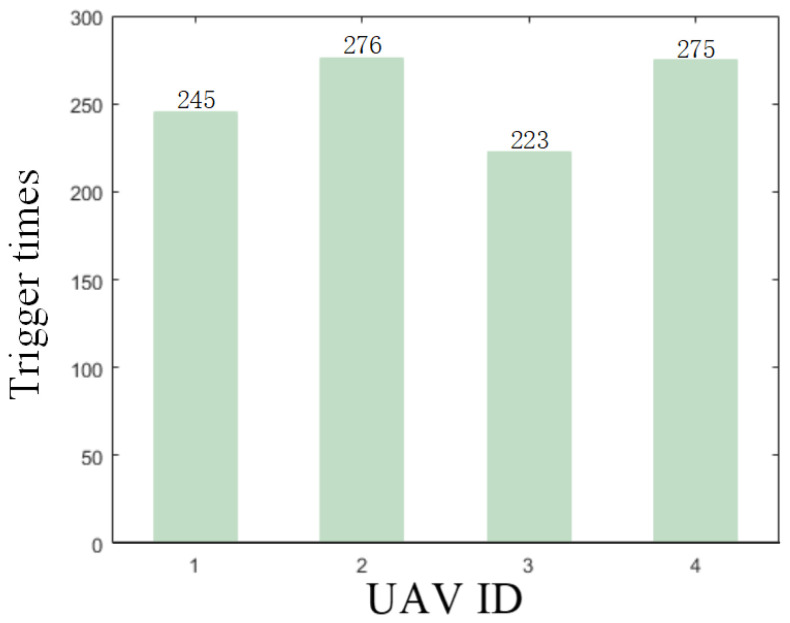
Scenario II: trigger times.

The simulation results are shown in Figure 14, Figure 15, Figure 16, Figure 17, Figure 18, Figure 19, Figure 20, Figure 21 and Figure 22. Figure 14 illustrates the enclosing trajectory under the equidistant rotating formation. It can be observed that the UAVs maintain the desired inter-agent distance and successfully form a stable rotating enclosing ring, even in the presence of communication delays. Figure 15 depicts the relative distance between the virtual leader and the target, which converges asymptotically to zero, confirming that the enclosing task is accomplished reliably despite delayed information exchange. Figure 16 and Figure 17 present the UAV velocity profiles and formation position error, respectively. The velocity curves converge smoothly, and the position error diminishes rapidly after initial adjustment, indicating that the system retains good dynamic response and steady-state performance under delayed communication. Figure 18 further demonstrates consistent speed tracking across all UAVs. The phase distribution in Figure 19 confirms that the UAVs maintain approximately equal angular spacing during enclosing, satisfying the design requirement of an equidistant rotating formation, even with communication delays. The evolution of the internal dynamic variable in Figure 20 shows a trend similar to that in the delay-free case, verifying that the proposed event-triggering mechanism remains effective under time-delay conditions. Finally, the event-triggering distributions and counts in Figure 21 and Figure 22 indicate that the strategy continues to significantly reduce communication frequency and alleviate network burden while preserving enclosing accuracy and formation stability in delayed environments.

### 4.3. Variable Range Rotating Formation to Encircle Maneuvering Target

To validate the algorithm’s scalability and the multi-UAV capability to encircle moving targets, this section presents an encirclement scenario with eight UAVs deployed against the target. Set targets in the form of segmented maneuvers, in 0–12 s, $u_{t}\left(t\right)={\left[3\cos t,3\sin t\right]}^{T}$; in 13–60 s, $u_{t}\left(t\right)={\left[4\sin t,4\cos t\right]}^{T}$. The initial phase angle is $\left[\pi ,\frac{\pi }{2},\frac{3\pi }{2},0,\frac{2\pi }{3},\frac{4\pi }{3},\frac{\pi }{4},\frac{3\pi }{4}\right]$ and the subsequent update of phase angle satisfies (15). In 0–30 s, the enclosing radius is $d_{i}=50$, and the rotational angular velocity ω=0.4; in 31–50 s, the enclosing radius is $d_{i}=40$, and the rotational angular velocity is ω=0.5; in 0–30, the enclosing radius is $d_{i}=30$, and the rotational angular velocity is ω=2/3.

**Figure 23:**
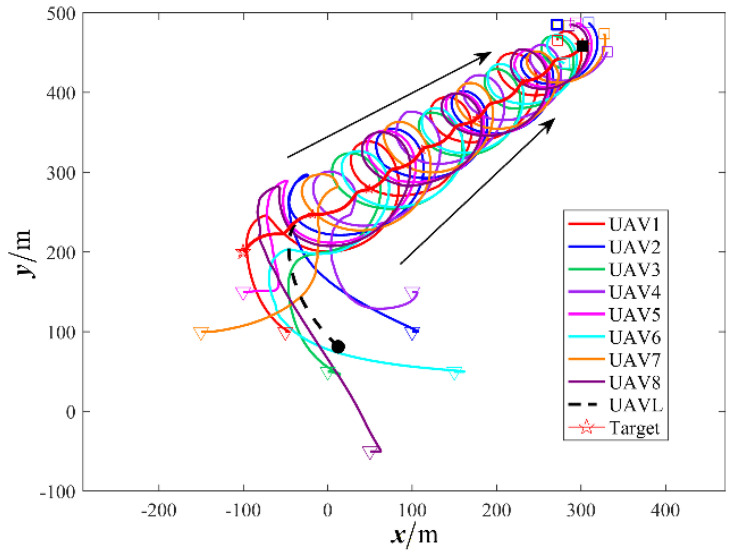
Scenario III: variable−spacing UAV formation trajectory for encircling a constant−velocity.

**Figure 24:**
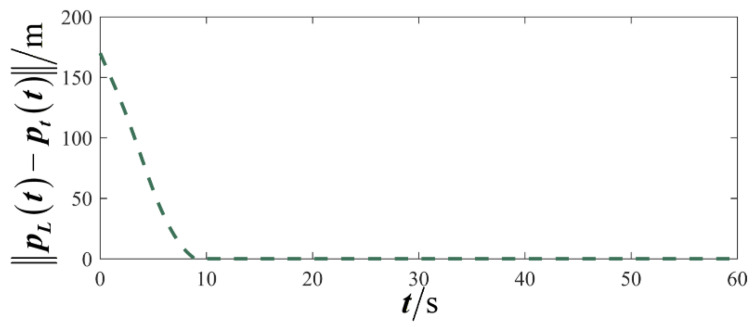
Scenario III: relative distance between virtual leader and target.

**Figure 25:**
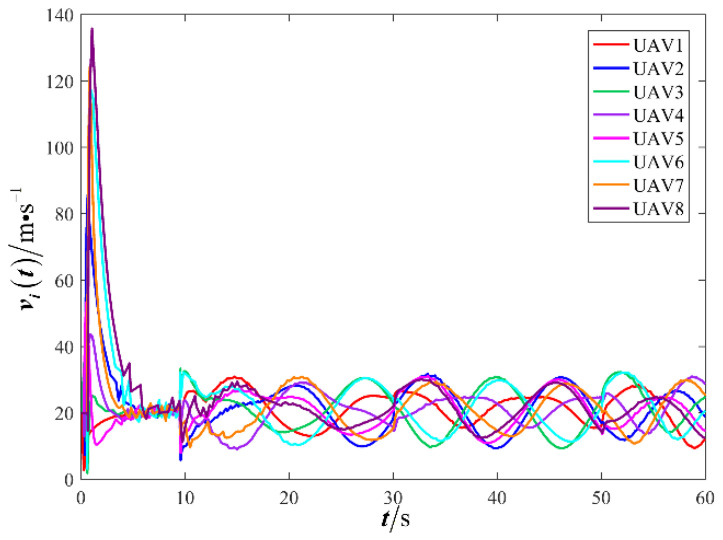
Scenario III: changes in UAV velocity.

**Figure 26:**
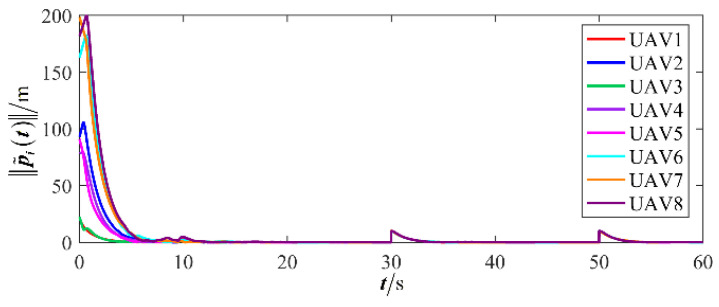
Scenario III: formation position error.

**Figure 27:**
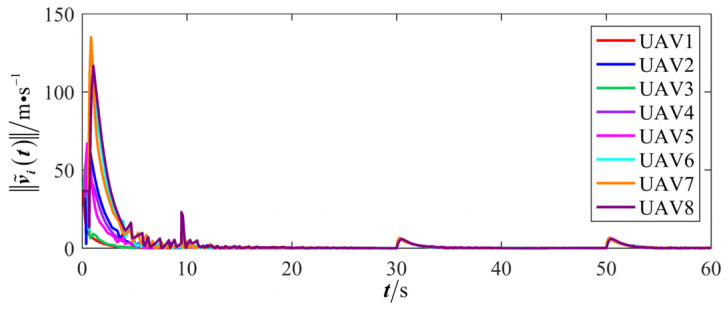
Scenario III: formation velocity error.

**Figure 28:**
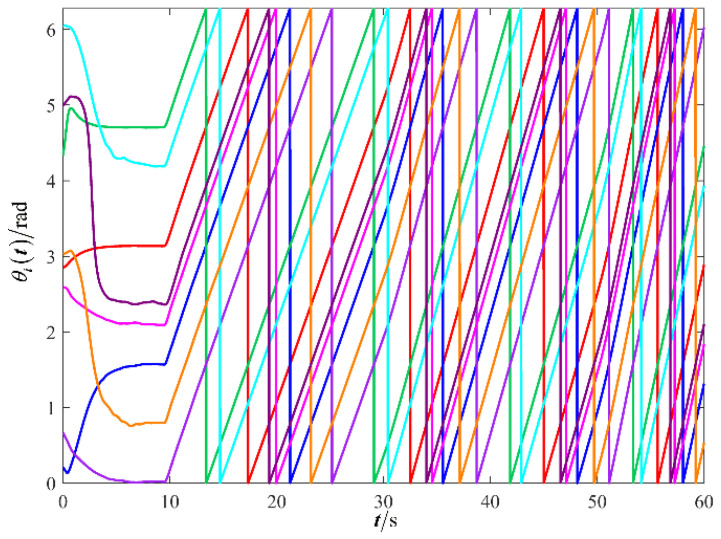
Scenario III: the phase of the UAV relative to the target.

**Figure 29:**
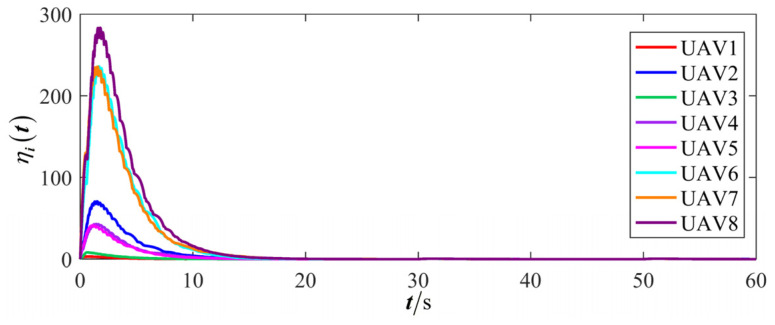
Scenario III: dynamic variable.

**Figure 30:**
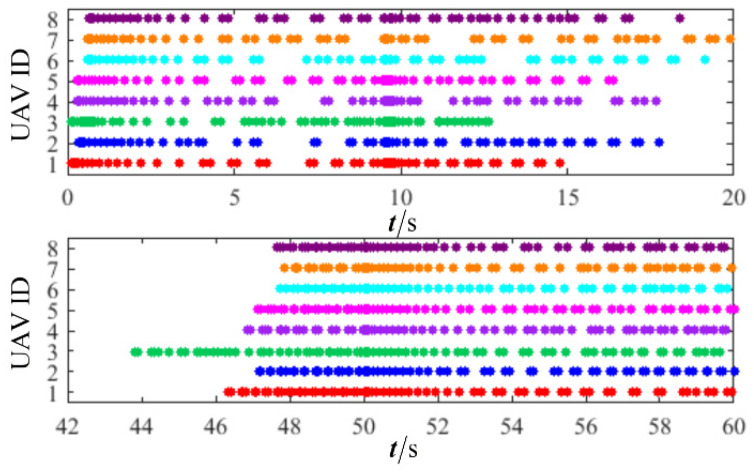
Scenario III: trigger distribution.

**Figure 31:**
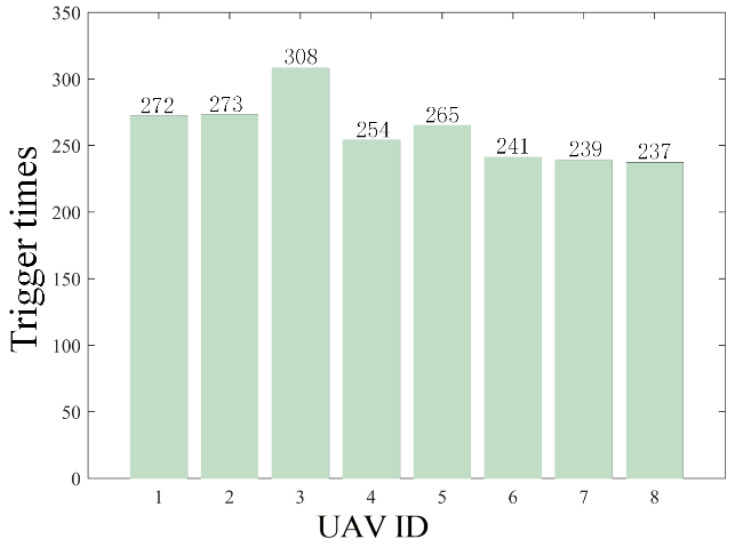
Scenario III: trigger times.

The results are displayed in Figure 23, Figure 24, Figure 25, Figure 26, Figure 27, Figure 28, Figure 29, Figure 30 and Figure 31. Figure 23 shows the enclosing trajectory of a maneuvering target by a UAV in a variable range rotating formation. Despite the continuous maneuvering of the target, UAVs can still adjust its formation in real-time and complete enclosing, reflecting this control strategy has good adaptability to maneuvering targets. The relative distance between virtual leader and target in Figure 24 still converges to zero, indicating that the system has good robustness to target maneuvering. Figure 25 and Figure 26, respectively, show the changes in the position error of UAV velocity and formation. In the stages of target maneuvering and changes in the radius of the enclosing ring, there is a brief increase in error. The controller can respond quickly and restore stability, demonstrating good disturbance suppression capability. The speed error in Figure 27 further indicates that each UAV maintains good consistency tracking performance in dynamic environments. The phase distribution diagram shown in Figure 28 indicates that the UAV can still maintain the expected phase structure under variable ranges, verifying the rationality of the formation factor design. The changes in dynamic variables in Figure 29 are consistent with the performance of the triggering mechanism, indicating that the mechanism can still work effectively in complex scenarios. The statistical results of event-triggering in Figure 30 and Figure 31 indicate that although the target has maneuverability, the drone formation can still achieve intermittent communication through dynamic event-triggering mechanisms, reduce communication pressure, and ensure the completion of the enclosing task.

### 4.4. Comparison and Analysis

**Figure 32:**
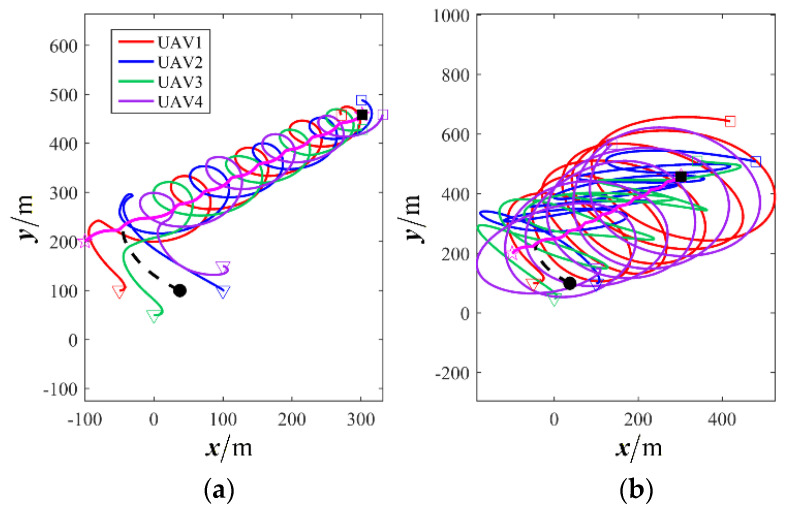
Comparison of target enclosing trajectories. (**a**) Proposed method; (**b**) comparative method.

**Figure 33:**
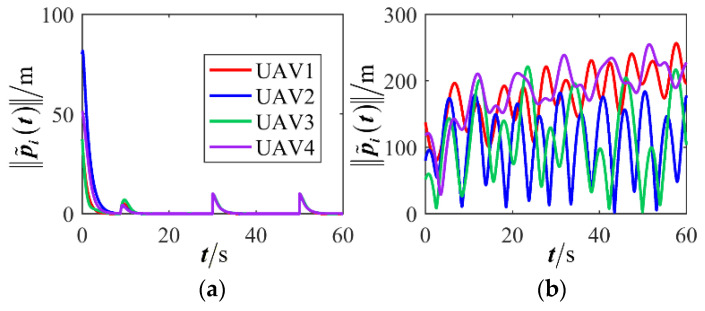
Comparison of formation position error. (**a**) Proposed method; (**b**) comparative method.

**Figure 34:**
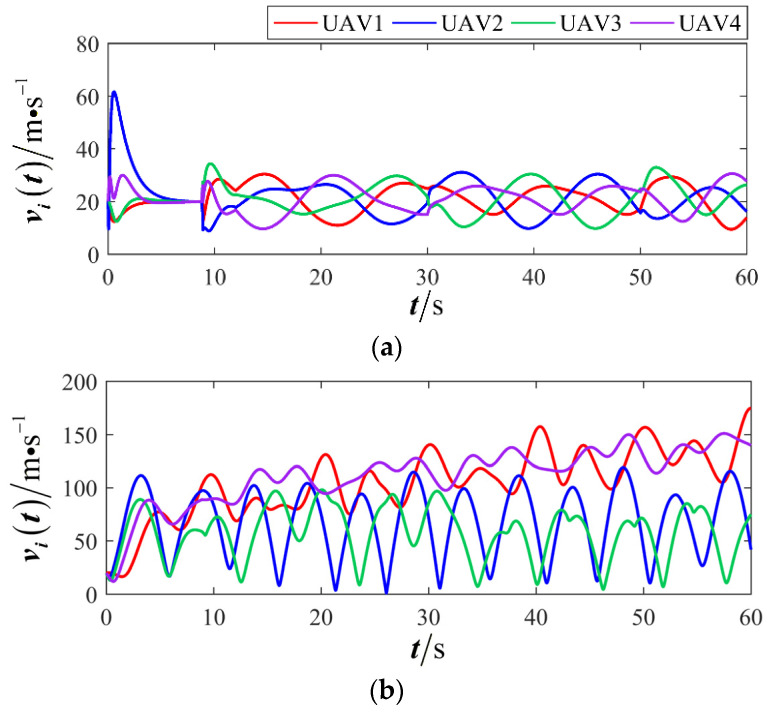
Comparison of UAV velocity. (**a**) Proposed method; (**b**) comparative method.

**Figure 35:**
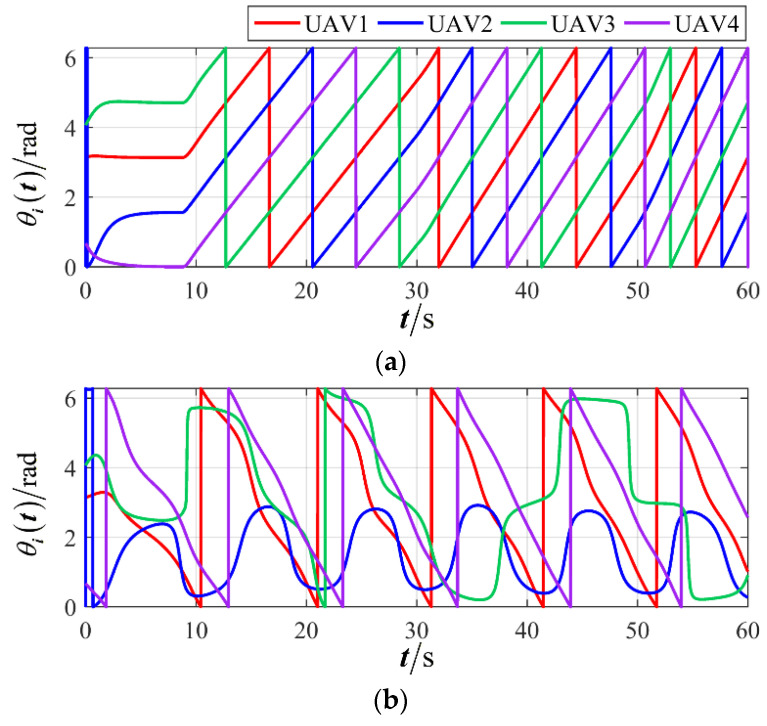
Comparison of UAVs’ phase. (**a**) Proposed method; (**b**) comparative method.

First, a comparative analysis with the target enclosing method presented in reference [26] is conducted to verify the effectiveness and superiority of the proposed control framework. The simulation results are depicted in Figure 32, Figure 33, Figure 34 and Figure 35. Figure 32 and Figure 33 illustrate a rotating formation capture scenario targeting a maneuvering object. It is demonstrated that the proposed method successfully establishes a stable and reliable encirclement configuration. In contrast, the approach from reference [26] fails to adapt effectively to the demands of a variable-distance rotating capture strategy, resulting in divergent UAV trajectories and growing formation errors. Figure 34 and Figure 35 provide further insight by, respectively, presenting the flight velocity of each UAV and its phase relative to the target. These figures confirm that the proposed strategy ensures satisfactory coordination among the UAVs, maintaining consistent velocity and stable phase relationships throughout the capture maneuver.

**Figure 36:**
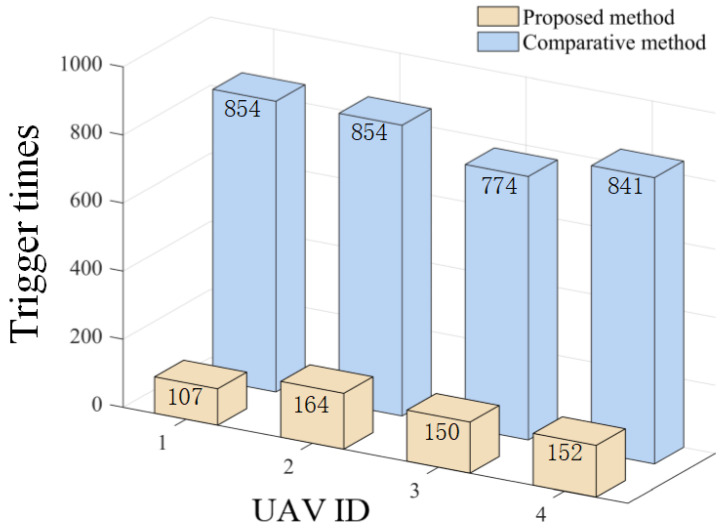
Comparison of trigger times at Scenario I.

**Figure 37:**
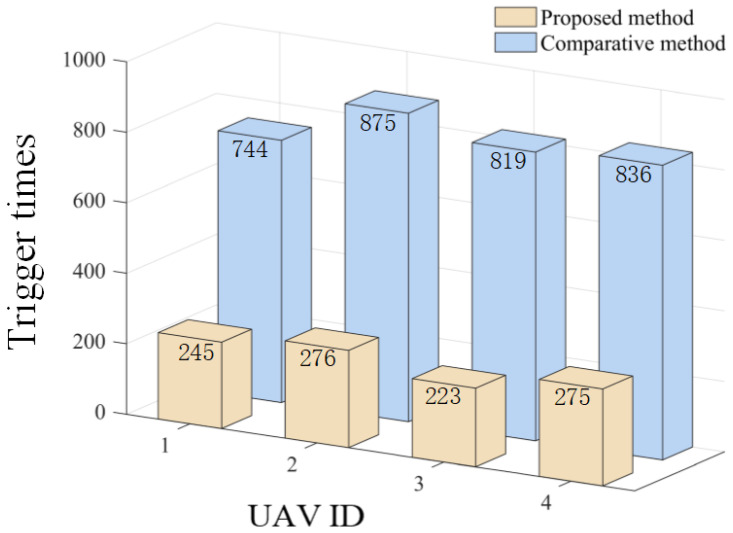
Comparison of trigger times at Scenario II.

**Figure 38:**
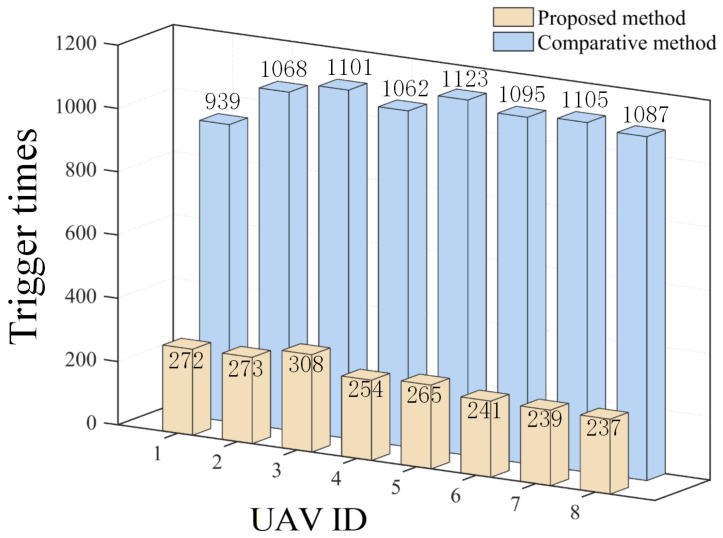
Comparison of trigger times at Scenario III.

The effectiveness of the proposed triggering mechanism is verified by comparing it with the method in [27]. Figure 36, Figure 37 and Figure 38, respectively, compare the triggering frequency of dynamic event-triggering strategy and static-triggering strategy in different scenarios. It can be seen that the dynamic event-triggering strategy can significantly reduce the quantity of triggers in all scenarios. The static-triggering strategy results in a significant communication burden due to continuous triggering. The dynamic event-triggering mechanism achieves adaptive adjustment of the triggering threshold by introducing internal dynamic variables, thereby significantly reducing communication frequency and computational load while ensuring control accuracy. The comparative results verify a significant strength of the presented approach in saving communication resources, which is suitable for practical engineering applications with limited resources.

## 5. Conclusions

This paper investigates the target enclosing control in UAV formation under communication resource constraints. A formation description method based on geometric transformation parameter set is proposed to overcome the shortcomings of traditional relative description methods in formation transformation. We have constructed an adaptive cooperative target enclosing architecture, achieving integrated design of formation control and target enclosing. In addition, a dynamic event-triggered control strategy is designed, which introduces internal dynamic variables to adaptively adjust communication triggering conditions, effectively reducing the system’s communication load while ensuring control accuracy. Through rigorous stability analysis and multiple simulation experiments, the effectiveness of this control strategy has been verified, and it has demonstrated superior performance in communication efficiency compared to static-triggering strategies. Future research will focus on the robustness of algorithms under non-ideal conditions such as wind disturbances in 3D and promote experimental verification of algorithms on physical UAV platforms.

## Figures and Tables

**Table 1:** Convergence times under different initial values.

$\eta_{i}\left(0\right)$	0.01	0.05	0.1	0.15	0.5	1.0
**Convergence time**	14.63 s	14.27 s	11.72 s	12.53 s	14.16 s	15.97 s

## Data Availability

Data supporting the findings of this study are available from the corresponding author upon reasonable request.
